# One-Step Confined Polymerization of Catecholamine Biopolymers for the Patterned *In Situ* Growth of Plasmonic Metasurfaces with Single-Particle Resolution

**DOI:** 10.1021/acsami.6c05739

**Published:** 2026-06-01

**Authors:** Serena Schiavi, Simone Ventisette, Pau Vilches Rueda, Maria Minunni, César Moreno, Angelo Taglietti, Simona Scarano, Leonardo Scarabelli

**Affiliations:** † Department of Chemistry, University of Pavia, Pavia 27100, Italy; ‡ Department of Chemistry “Ugo Schiff’, 9300 University of Florence, Sesto Fiorentino 50019, Italy; § NanoOddLAB, Department of Chemistry and Process & Resource Engineering, ETSIIT, 16761 University of Cantabria, Santander 39005, Spain; ∥ Department of Pharmacy, 9310 University of Pisa, Pisa 56126, Italy; ⊥ Departamento de Ciencias de la Tierra y Fisica de la Materia Condensada, Universidad de Cantabria, Santander 39005, Spain

**Keywords:** plasmonic metasurfaces, catecholamine biopolymers, in situ growth, bottom-up synthesis, patterned growth

## Abstract

Patterned *in situ* growth of plasmonic nanostructures is emerging as a rational bottom-up route to fabricating functional plasmonic metamaterials. In this family of approaches, the combination of simple wet-chemistry principles with tailored surface-chemistry modifications unlocks new, scalable, and versatile strategies to produce ordered nanoparticle arrays showing strong electromagnetic responses. Herein, the self-polymerization of catecholamine monomers is exploited to achieve region-specific chemical contrast via a one-step polymer patterning approach that is compatible with different materials and can be completed in a few minutes. To highlight the robustness and versatility of this methodology, the confined polymerization protocol was successfully applied across multiple length scales, from micrometer-sized patterns to nanometric arrays with feature sizes down to a few tens of nanometers, enabling the simultaneous fabrication of multiple patterns on a single substrate within the same preparation. The obtained chemically patterned substrates were exploited to control the gold nanoparticle nucleation point with nanometric precision, leading to the growth of plasmonic arrays. The size of the gold nanoparticles and the number of particles per polymer-patterned feature can be controlled by tuning the growth conditions, underscoring the power of bottom-up wet-chemical *in situ* approaches for the scalable fabrication of tunable plasmonic architectures showing single-particle resolution (i.e., single nucleation for each active area). The obtained plasmonic metasurfaces exhibit lattice plasmon resonances in the visible and near-infrared spectral regions, reaching quality factors as high as 130. Overall, these results pave the way for advanced sensing, catalytic, and other nanophotonic applications requiring large-area, highly ordered arrays.

## Graphical abstract

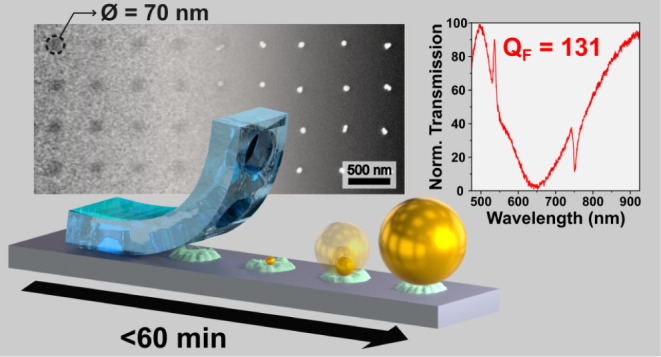

## Introduction

Localized surface plasmon resonances originate from the collective oscillation of the conduction band electrons of noble-metal nanoparticles upon interaction with an electromagnetic field.^1,2^ This interaction gives rise to peculiar nanoscale phenomena that have found application in a variety of fields, such as the generation of high-energy carriers for catalysis and optoelectronics, localized heat for photothermal therapies, or intense electric fields that can be exploited for sensing, nanophotonics, and nano-optics.^3–6^ The precise engineering of these plasmonic effects relies on tight control over nanostructure size, morphology, crystallography, and surface chemistry, representing a major driving force for the development of ever more optimized wet-chemical synthetic strategies. As a result, over the last three decades, shape yields above 90% and size dispersities below 10% have been achieved for a variety of particle sizes, shapes, and compositions.^7–9^

However, the integration of nanocolloids into functional solid-state devices without compromising their physicochemical properties remains challenging. Colloidal self-assembly requires several additional steps and specifically designed ligands, which significantly limit scalability and versatility, while top-down lithography relies on serial, time-consuming, and expensive writing processes that severely limit high-throughput production. Furthermore, metal deposition techniques typically employed in lithography yield polycrystalline structures with high resistive losses, failing to provide control over surface chemistry and crystallography. As such, *in situ* growth has recently emerged as a more direct and promising way to develop functional plasmonic devices, where the growth of plasmonic nanostructures takes place directly on the desired surface. Nonetheless, *in situ* growth has remained a relatively unexplored branch of bottom-up colloidal synthesis, mainly because of a general lack of control over crystal nucleation and development.^10^

Very recently, significant steps forward in the field have brought new light and attention to this approach. Examples include the work of Piella et al., who introduced a regioselective sacrificial polymer mask that is etched to expose defined seed surface fractions.^11^ Despite developing a hybrid method still relying on top-down lithography, this work demonstrates the high-yield growth of anisotropic palladium nanostructures *in situ*. More recently, Lonza et al. achieved up to 90% shape yield of anisotropic gold nanorods and nanostars grown directly within functionalized microfluidic channels, bypassing any colloidal seeding step and enabling precise growth control via reagent flow and surface chemistry.^12^ This contribution demonstrated how nanostructures with comparable yield and quality to colloidal systems can be prepared *in situ,* even for complex shapes like single-crystal gold nanorods, which require both kinetic and thermodynamic control.^13^ Finally, Vinnacombe-Willson et al. optimized an *in situ* seeded growth method for plasmonic gold nanostars on biocompatible gelatin-based hydrogels.^14^ By exploiting intrinsic polymer–metal interactions and surfactant-free conditions suitable for biological applications, this work shows how *in situ* growth can be extended beyond standard substrates to include soft materials and 3D structures.

However, in order to express *in situ* growth’s full potential, there is still a key missing element: i.e., the capability to predetermine the surface nucleation point of each nanoparticle with nanometric precision. In fact, such patterned *in situ* growth approaches would enable control over the reciprocal spatial distribution (and potentially the orientation) of the plasmonic units, resulting in a direct synthetic route for plasmonic metasurfaces and metamaterials. In these devices, the ordered placement of metal nanoparticles with a period comparable to the wavelength of the incident light produces diffracted waves that promote the far-field coupling between the localized plasmon resonances of the individual nanoparticles.^15,16^ Under these conditions, a reinforcement of the resonance in the neighboring particles is achieved, which, extended over a large array, leads to the emergence of collective lattice plasmonic resonances (or surface lattice resonances, SLRs) that are characterized by the drastic reduction of the plasmon bandwidth, ultimately translating into more than a 10-fold increase in the associated quality factors (*Q*_f_).^15,16^

Metasurfaces’ sharp optical features can have a major impact in boosting the sensitivity of plasmon-based optical detection methodologies, while their long-lived excited states can be extremely important for photocatalysis and optoelectronic devices. Control over the metasurfaces’ physicochemical properties, offered by patterned *in situ* growth, could have a transformative impact on these applications. The most common methodologies for the fabrication of metasurfaces composed of chemically grown nanostructures rely on colloidal self-assembly,^17–19^ microcontact printing,^20,21^ dynamic templating,^22–24^ and solvent-assisted nanoscale embossing (SANE).^25^ In 2022, we demonstrated that chemical patterning could generate two surface regions presenting a stark chemical contrast, resulting in the selective nucleation and growth of gold nanoparticles only on one of the two regions.^26^ This chemical contrast was leveraged to obtain plasmonic metasurfaces using a simple wet-chemistry growth. Although the emergence of SLRs with no colloidal synthesis or top-down fabrication steps was a major stepping stone for validating *in situ* growth as a viable nanopatterning process, the procedure requires multiple soft-lithography steps, resulting in long processing times and sample-to-sample variability, while strongly limiting the types of compatible substrates on which the protocol could be carried out.

Herein, we introduce a simple one-step approach using the confined polymerization of dopamine (DA) and norepinephrine (NE) under a PDMS template to generate the necessary surface chemical contrast, enabling the robust and scalable *in situ* growth of plasmonic arrays on different substrates within an hour. Catecholamine biopolymers are biomimetic materials that, over the past two decades, have found extensive applications in several research fields because of their remarkable multifunctional properties as coating materials.^27,28^ The primary advantage of these biopolymers is their straightforward and effective preparation. Exploiting the solution oxidation method, nanometric polydopamine (PDA) and polynorepinephrine (PNE) layers are achieved by the self-polymerization of the monomers in weak alkaline media in the presence of oxygen.^29,30^ Notably, this polymerization process proceeds under mild, water-based conditions without the need for any additional oxidant, yielding intrinsically biocompatible and environmentally sustainable materials.^31^ Furthermore, by controlling key parameters such as polymerization time and monomer concentration, the thickness of the deposited biopolymer layers can be precisely tuned at the nanometer scale (typical thickness values range between 2 and 50 nm).^32^ Another key advantage of catecholamine-based biopolymers is their ability to adhere to most organic and inorganic substrates.^29^ Moreover, thanks to the many functional groups that PDA and PNE incorporate (including catechols, amines, and imine groups), these biopolymers present a rich chemistry that includes covalent modifications, ionic coordination, and redox chemistry.^33–38^

This one-step solution-based approach yields reactive biopolymer patches by confining the catecholamine polymerization using a PDMS stamp. The protocol was successfully implemented for patterns presenting feature sizes from 70 nm to several micrometers, even within the same preparation (i.e., at the same time and on the same substrate). Subsequent selective nanoparticle growth was achieved via a seed-mediated process, enabling precise control over particle density while preventing secondary nucleation from taking place in the growth solution away from the substrate. To assess the versatility of the procedure, the growth was replicated on silicon (crystal-flat surface), glass (amorphous and rough surface), and PDMS (elastomer). The resulting periodic arrays sustain ultrasharp SLRs with *Q*_f_ up to 131, among the highest reported to date in the visible range. Overall, these results demonstrate how catecholamine-driven patterned *in situ* growth provides a straightforward and scalable route to engineer highly delocalized SLRs with profound long-term implications for sensing, catalysis, and photonic devices.

## Results and Discussion

### Biopolymers Patterning by Confined Polymerization

The patterned *in situ* growth of gold nanoparticle arrays is driven by the chemical contrast between the bare substrate and two biopolymers, PDA or PNE. The chemically patterned substrates were fabricated using an elastomeric nanostructured stamp to confine the spontaneous DA and NE self-polymerization in an alkaline aqueous solution. The procedure is schematized in Figure 1A. For a standard preparation, 2 μL of a 2 mg/mL NE or DA solution buffered at pH 8.5 in 10 mM Tris-HCl is drop-cast onto a clean 1 × 1 cm^2^ substrate and rapidly covered with a hard polydimethylsiloxane (hPDMS) mold featuring the desired pattern. A pressure of approximately 0.5 bar is applied for 10 min, after which the hPDMS mold is gently removed. Polymerization time and pressure were optimized using a combination of Scanning Electron Microscopy (SEM) and Atomic Force Microscopy (AFM). After demolding, the patterned substrate is rinsed with Milli-Q water and dried under a nitrogen stream before further characterization (more details on the patterning process and its optimization are reported in Supporting Information, S1–S2). When silicon is used as the substrate, the successful polymer patterning was confirmed by SEM for both PNE and PDA (Figure 1C and D, respectively). The resulting circular biopolymer patches exhibited smaller diameters than those of the corresponding hPDMS mold cavities. For instance, a mold with a 500 nm periodicity (Λ) and 230 nm cavity diameter (Ø) (Figure 1B) yielded polymeric patches with an average diameter of 180 ± 10 nm (Figure 1C and D). Possible causes of this contraction may be related either to the demolding step, during which the PNE/PDA films attached to the PDMS sidewalls could be partially detached upon stamp removal, or to the compression applied during the imprinting process. Even though, at present, we do not have direct experimental evidence to unambiguously identify the origin of this phenomenon, this contraction had important consequences for the subsequent gold nanoparticle growth step, which will be discussed in the next section of the manuscript; the full data comparing cavity dimensions of the hPDMS stamp with synthesized polymer patches are reported in Supporting Information, S3. Counterintuitively, the images acquired by Secondary Electron Detection evidence dark spots corresponding to the polymerized regions, suggesting a subnm thickness causing a drop in the secondary electrons detected from the silicon substrate. Although both DA and NE gave identical results, PNE has recently attracted increasing interest because it can yield smoother and more uniform films, potentially offering improved control over the resulting polymer coating.^31^ For this reason, the remaining part of the text will focus on the use of PNE, while PDA results will be reported in the Supporting Information.

**Figure 1:**
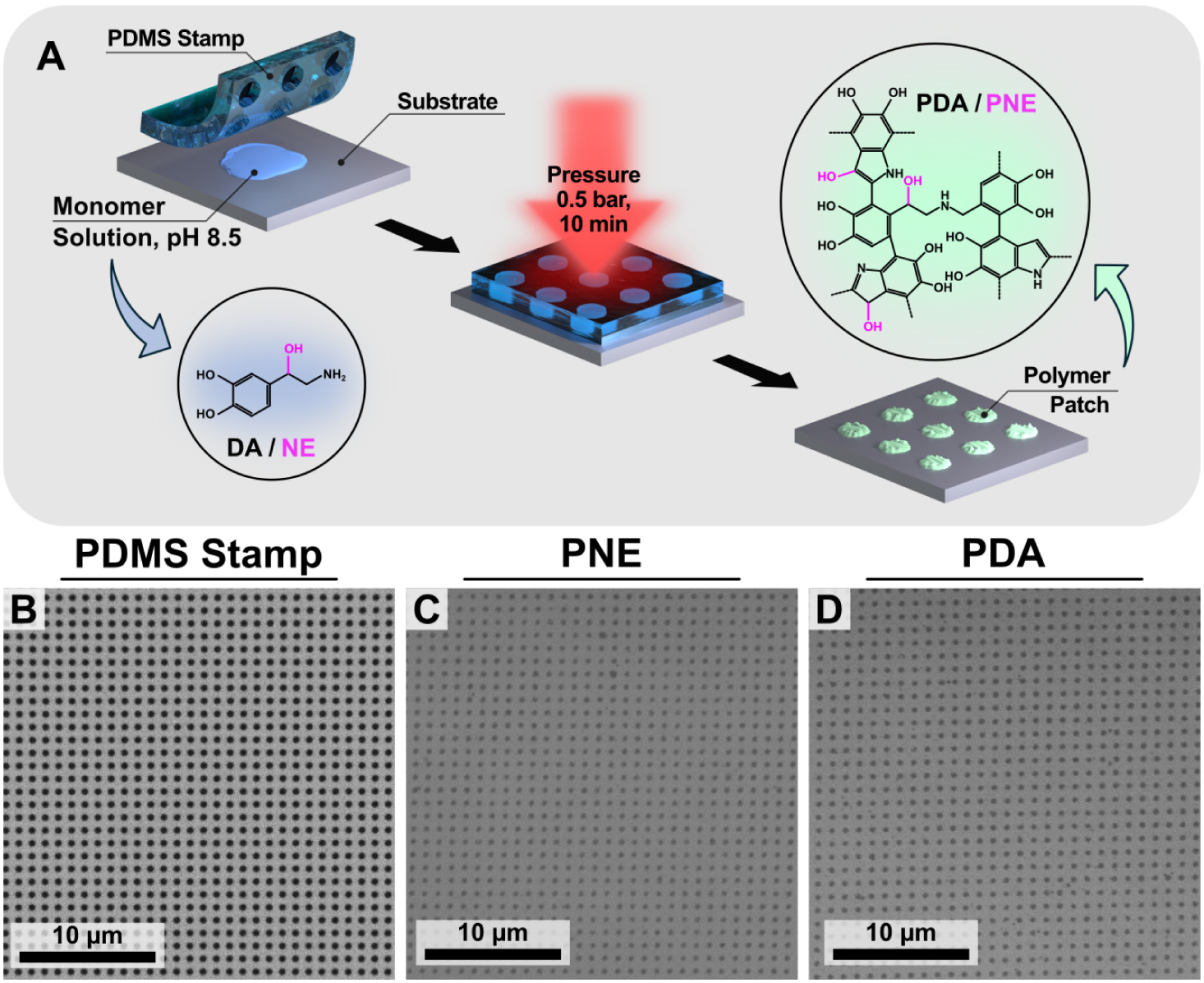
PNE/PDA confined polymerization. **A**: Schematic of the PDA and PNE confined polymerization patterning process. **B:** Backscattered electron SEM image of a square hole array hPDMS stamp with a periodicity of 500 nm. **C, D:** Secondary electron SEM images of the PNE (**C**) and PDA (**D**) patterns obtained after 10 min of confined polymerization over a silicon substrate, applying 0.5 bar of pressure.

One of the key advantages of the developed patterning protocol lies in its scalability and versatility, enabling the fabrication of multiple biopolymer patterns with distinct periodicities and dimensions. To test its scalability, we fabricated a 5 × 5 cm^2^ hPDMS stamp featuring 9 different patterns (Figure 2A; detailed procedure reported in the Supporting Information, S2.7). The standard procedure described was then simply adapted to a multiarray configuration: the monomer solution (NE or DA) was drop-cast onto the multiarray hPDMS stamp, with one 2 μL drop for each 1 × 1 cm^2^ patterned region, followed by a clean silicon wafer and the application of a pressure of 0.5 bar. Successful PNE patterning was achieved in a single step across all 9 arrays (Figure 2B–J), highlighting the robustness and scalability of the method. Notably, the self-polymerization process can be easily adapted to patterns of various dimensions, spanning from micrometric spirals and lines (Figure 2I, J) to nanometric square arrays with lateral dimensions from Ø of 180 nm (Figure 2B) down to Ø of 70 nm (Figure 2H). Moreover, the same strategy was effectively extended to DA, allowing the preparation of PDA-based reactive patterns using identical synthetic conditions (comprehensive SEM images are reported in Supporting Information, S4).

**Figure 2:**
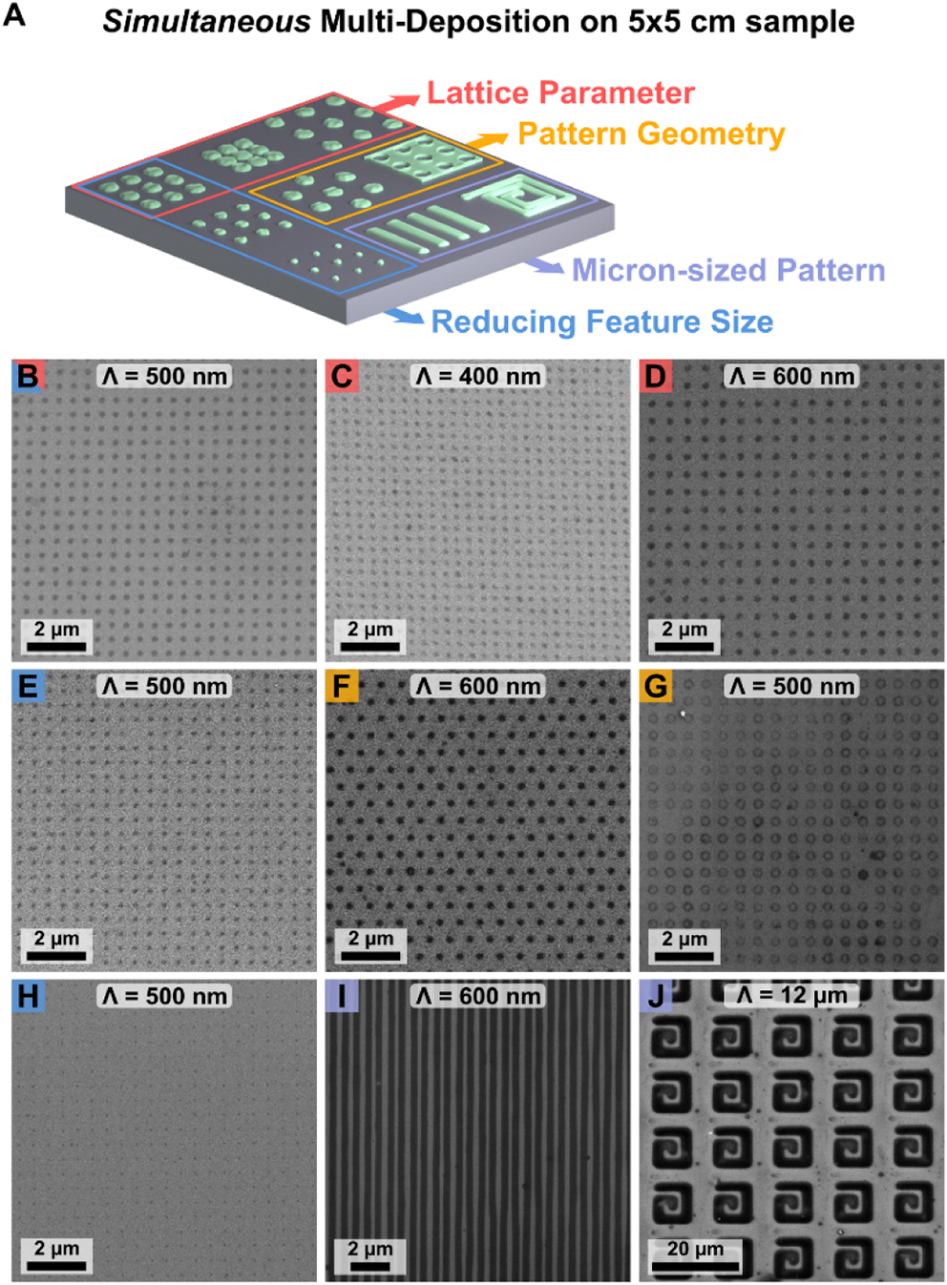
Scalability of the methodology for simultaneous multipattern deposition. **A:** Schematic of a 5 × 5 cm^2^ multipatterned sample prepared in a single confined polymerization step. **B–J:** Secondary electron SEM images of all the PNE-patterned regions prepared simultaneously over the sample: square patterns with a periodicity (Λ) of 400 nm (**B**), 500 nm (**C**) and 600 nm (**D**); square patterns with reduced patch dimensions (Ø) of 120 (**E**) and 70 nm (**H**); hexagonal pattern with a Λ of 600 nm (**F**); pillar pattern with a Λ of 500 nm (**G**); line pattern with a Λ of 600 nm (**I**); and spiral pattern with a Λ of 1500 nm (**J**).

The obtained polymeric patterns of both PNE and PDA were characterized using AFM (Figure 3). Phase-contrast AFM images, which are commonly associated with variations in energy dissipation at the tip–sample interface, revealed localized changes in surface properties such as friction, adhesion, viscoelasticity, and compositional heterogeneity. These differences identify a clear contrast in mechanical properties between the silicon substrate and the PNE and PDA patches (Figure 3A–D, respectively). AFM topography profiles (Figure 3B–E) allowed the determination of an average patch thickness of 0.8 ± 0.2 nm after 60 min of confined polymerization. For both PNE and PDA, the average thickness value was calculated from 100 measurements collected across multiple samples by subtracting valley heights from the corresponding peak heights (Figure 3C–F). Notably, according to previous reports, solution oxidation under comparable conditions typically yields catecholamine-based biopolymer films with significantly higher thicknesses (typically 4–6 nm after 60 min).^39^ We therefore hypothesize that the confinement of a few microliters of monomer solution beneath a ∼0.5 cm-thick PDMS stamp hinders oxygen exposure and diffusion, thereby slowing down or even arresting the polymerization process.^40^ To gain further insight into the reaction kinetics, AFM analysis was repeated for PNE-patterned substrates obtained after shorter polymerization times (phase-contrast and topography images reported in Supporting Information, S2.4). The results revealed average film thicknesses of 0.7 ± 0.2 nm and 0.9 ± 0.2 nm after 10 and 30 min of polymerization, respectively, suggesting that reduced reaction times do not lead to a significant decrease in film thickness. This behavior is consistent with the hypothesized rapid and self-limiting polymerization process under confined conditions. Based on these results, the optimal polymerization time for both PNE and PDA patterning was set to 10 min. Minor protocol adjustments were employed for larger arrays, such as spirals and lines (all details can be found in Supporting Information, S2.6).

**Figure 3:**
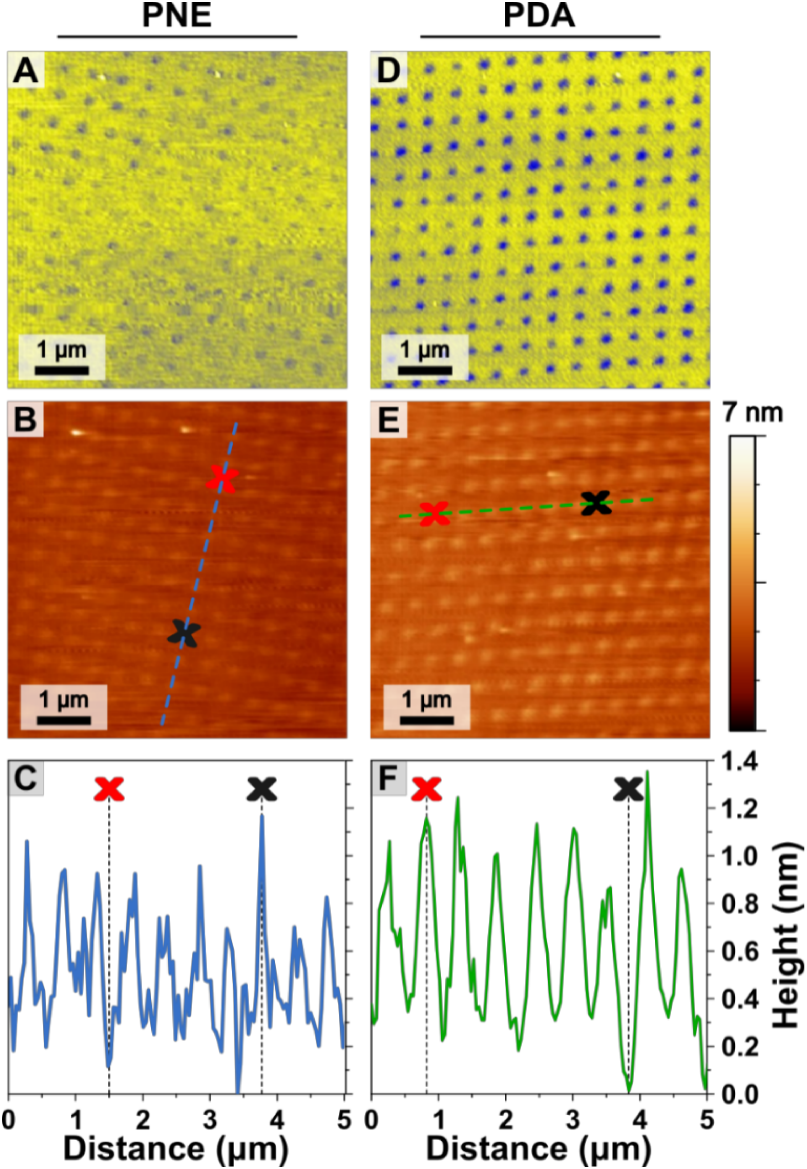
Polymer patch thickness. Phase-contrast AFM (**A, D**), topographic images (**B, E**), and extracted height profiles (**C, F**) of a PNE (**A-C**) and PDA (**D-F**) pattern on Si substrates with Λ = 500 nm after 60 min of polymerization.

### Patterned *In Situ* Growth of Gold Nanoparticle Arrays

After establishing optimal conditions for the fabrication of chemically patterned PDA and PNE substrates, we moved on to exploit the obtained chemical contrast for the patterned *in situ* growth of gold nanoparticle arrays. The main challenge of this process lies in the competition between gold nucleation directly on the substrate and secondary nucleation occurring in solution.^10^ Uncontrolled nucleation formation can severely hinder the success of *in situ* growth, as particles that form in solution away from the substrate would follow an alternative growth mechanism, resulting in different shapes, sizes, and crystallographies. Moreover, the products of secondary nucleation would deposit randomly, compromising the formation of an ordered nanoparticle array within the chemically patterned regions.^26^

To prevent uncontrolled growth, seed-mediated growth was adapted to the fabricated patterned polymeric substrate. In this type of approach, small nuclei are synthesized first, while avoiding the formation of new nuclei during growth.^41^ The seeding step was carried out through a two-stage process (Figure 4A). First, the chemically patterned silicon substrate was exposed to an aqueous gold precursor solution (HAuCl_4_) for 5 min, allowing Au^3+^ species to anchor onto the biopolymer surface (control experiments can be found in the Supporting Information, S5). Upon exposure to NaBH_4_, the surface-bound gold species were rapidly reduced, leading to the formation of nanometric seeds. Subsequent rinsing and quenching steps ensured the removal of residual reducing agent, yielding a clean and well-defined surface prior to the final growth stage. Even though PNE and PDA can be in principle exploited in the seeding step due to their intrinsic reducing capability, skipping the NaBH_4_ exposure step resulted in a slight but still significant increase in particle density (Supporting Information, S5). For this reason, the seeding step was finally performed through a NaBH_4_-mediated approach, which proved essential to achieve tight control over particle nucleation and final particle density, getting as close as possible to a single-particle metasurface. The successful formation of gold seeds was verified by AFM, since SEM imaging could not resolve such small gold nuclei. Compared to the same analysis previously performed on the polymer patterns, a subtle contrast variation was also observed within the patches themselves, suggesting local heterogeneity after gold seed formation (Figure 4B and Supporting Information, S6). AFM topographic images revealed an average thickness of the growth area of 2.1 ± 0.7 nm (Figure 4C and Supporting Information, S6), corresponding to an increase of approximately 1.4 nm compared to the untreated PNE polymer patches. This thickness increase is consistent with the presence of newly formed gold nuclei and agrees well with the typical dimensions of colloidal gold seeds synthesized in the presence of cetyltrimethylammonium bromide (CTAB, 1–2 nm) or citrate (3–5 nm).^42–44^ In the final growth step, 150 μL of a growth solution containing CTAB as the capping ligand, HAuCl_4_ as the gold precursor, and ascorbic acid as the mild reducing agent was drop-cast over the seeded substrate (more details on the procedure are described in the Supporting Information, S7).^26^ To avoid any secondary nucleation, the solution was rapidly discarded after 1 min, and the substrate was washed abundantly with Milli-Q water. The use of a weak reducing agent at this stage was essential: ascorbic acid was only responsible for the reduction of Au^3+^ to Au^+^, enabling a controlled enlargement of the preformed gold seeds, which are responsible of catalyzing the reduction of Au^+^ to Au^0^.^45^

**Figure 4:**
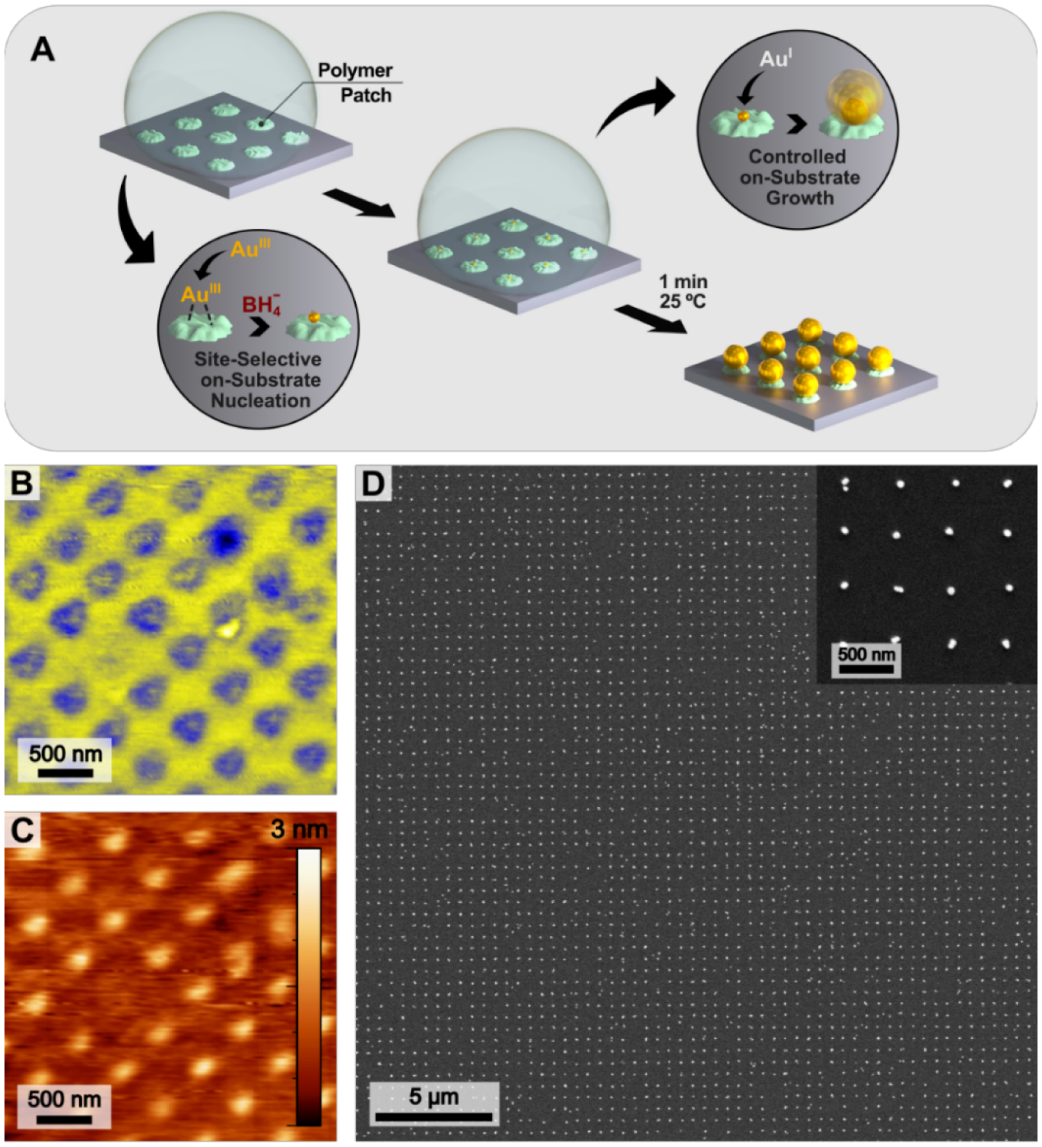
Seed-mediate *in situ* patterned growth. **A:** Schematic of gold nanoparticles’ *in situ* seed-mediated growth on PNE or PDA-patterned substrates. **B–C:** Phase-contrast AFM (**B**) and topographic images (**C**) of the PNE pattern (Λ = 500 nm) on a silicon substrate after the gold seeding step. **D:** Representative SEM image of the obtained gold nanoparticle array on a PNE pattern (Λ = 500 nm, Ø = 70 nm). **Inset:** High magnification SEM image enabling a better view of particle morphology and size distribution.

Pattern-selective particle growth was confirmed by SEM, revealing well-defined arrays of gold nanoparticles. The obtained particle arrays were mechanically stable and remained firmly attached to the substrate after extensive rinsing and drying (Figure 4D). Morphological analysis of the SEM images revealed an average diameter of the gold nanoparticles of 60 ± 10 nm.

The seeding and growth steps were systematically optimized to control the distribution, density, and size of the nanoparticles within the PNE/PDA patches. In particular, the number of particles growing within a single polymer patch was tuned by adjusting the concentration of Au^3+^ during the seeding stage. By reducing the Au^3+^ concentration from 0.5 mM to 0.02 mM, the average number of gold nanoparticles per patch decreased significantly from 4 ± 1 to 1.6 ± 0.8, while the fraction of single-particle patches increased from 1% to 46%, with overall patch coverage remaining nearly complete (from 100% to 96%, Figure 5A–B). It is important to highlight here that the average was calculated considering all the growth regions, including those showing no nucleation. Further lowering the Au^3+^ concentration led to an additional reduction in particle density (0.8 ± 0.8 particles per patch at 0.01 mM), translating to a slight increase in the fraction of single-particle patches to 48.5%, but at the expense of patch coverage, which dropped to 63% (Figure 5C and Supporting Information, S8). This optimization was performed on patches with a Ø of 180 ± 10 nm; by reducing the patch size, the statistics can be improved significantly, as discussed in the next section.

**Figure 5:**
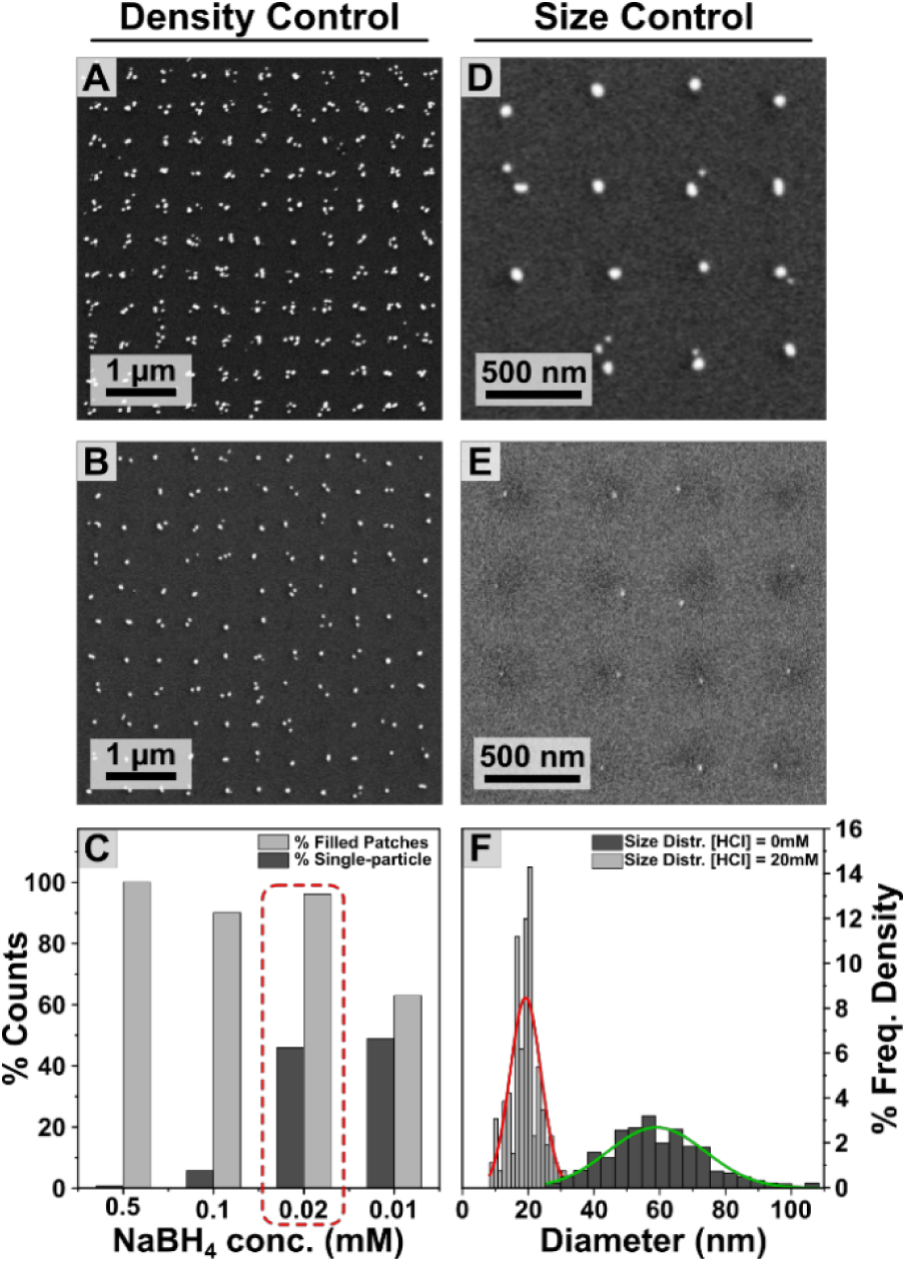
Controlling nanoparticle density and size. **A–C** Density control. **A–B:** SEM images of nanoparticle arrays obtained at [Au^3+^] = 0.5 mM (**A**) and 0.02 mM (**B**) during the seeding step. **C:** Percentage of filled patches (**light gray columns**) and single-particle patches (**dark gray columns**), with the optimal configuration highlighted (**red dashed box**). **D–F** Size control. **D–E:** SEM images acquired at [HCl] = 0 mM (**D**) and 20 mM (**E**), showing size tuning during the growth step. **F:** Corresponding nanoparticle size distributions ([HCl] = 0 mM, **dark gray columns, red line**; [HCl] = 20 mM, **light gray columns, green line**). Additional SEM images can be found in the Supporting Information, S9.

Nanoparticle size can, in principle, be tuned by modifying the growth time. However, growth times shorter than 1 min are hard to maintain consistently , while longer growth times exponentially increase the chance of secondary nucleation. Instead, nanoparticle size was controlled by varying the pH of the growth solution through the addition of hydrochloric acid. This strategy is commonly used in gold nanoparticle synthesis to modulate the pH of the reaction medium, which directly affects nucleation and growth kinetics. An acidic environment stabilizes Au^3+^ species while reducing the reduction potential of ascorbic acid, thereby influencing the reduction rate of Au^0^.^46^ By adding hydrochloric acid to a final concentration of 20 mM, the particle diameter was successfully reduced from 60 ± 10 nm to 19 ± 8 nm (Figure 5D–E and Supporting Information, S9).

Growth conditions yielding an average of 1.6 ± 0.8 (HAuCl_4_ 0.02 mM) nanoparticles per patch with an average diameter of 60 ± 10 nm (HCl 0 mM) were selected to ensure straightforward SEM characterization of the obtained metasurfaces while avoiding secondary nucleation. This protocol was successfully extended to different PNE and PDA pattern geometries (Figure 6A–D and Supporting Information, S10 and S11), enabling the fabrication of ordered plasmonic arrays, including square arrays with different lattice parameters, hexagonal arrays, and linear patterns.

**Figure 6:**
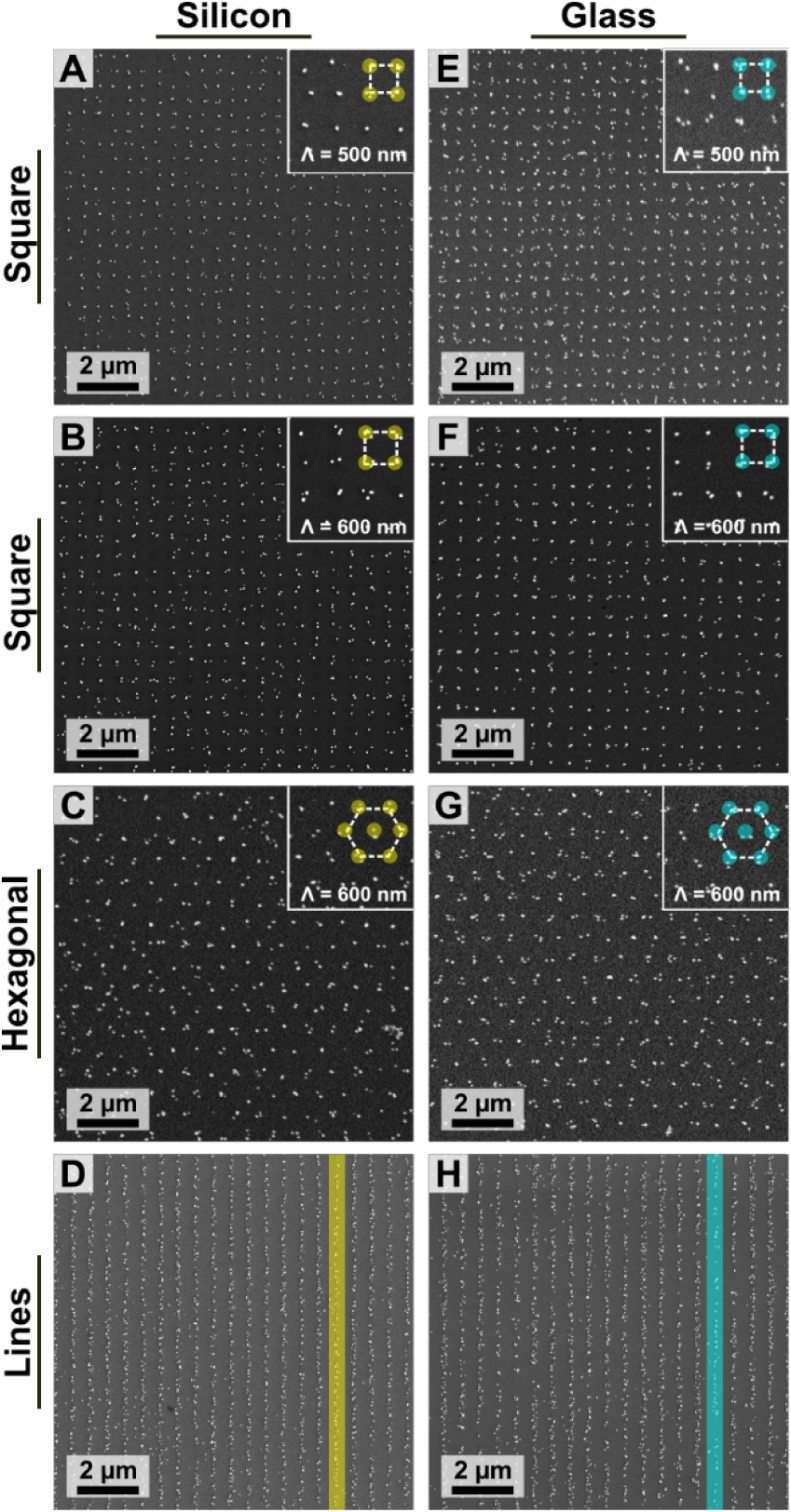
Modifying metasurface geometry and substrate. SEM images of nanoparticle arrays presenting different pattern geometries on different substrate materials: silicon (**A–D**) and glass (**E–H**). The tested patterns include square patterns with Λ = 500 nm, Ø = 180 nm (**A, E**), and Λ = 600 nm, Ø = 210 nm (**B, F**); hexagonal pattern with Λ = 600 nm, Ø = 200 nm (**C, G**); and line patterns with Λ = 600 nm (**D, H**). **Insets**: Higher-magnification SEM images where the unit cell is highlighted. Additional SEM images of all patterns can be found in the Supporting Information, S10–S11.

Another major advantage of the developed approach lies in its versatility when it comes to substrate compatibility, as both PNE and PDA have been deposited on a variety of materials, including metals and semiconductors, crystalline and amorphous substrates, and hydrophobic and hydrophilic surfaces.^47,48^ Apart from silicon, the fabrication was tested on glass and PDMS (Figure 6E–H and Supporting Information, S12). No significant differences were observed between substrates, either during the PNE/PDA polymerization or throughout the subsequent *in situ* gold-growth process. These findings further confirm the high versatility of the developed procedure, from the initial substrate modification step to the final formation of patterned plasmonic surfaces.

### Lattice Plasmon Resonance Characterization

The use of NE and DA self-polymerization readily allows tuning the diameter of the chemically reactive patterned patches, thus providing tighter control over the exact nucleation point, resulting in higher-quality metasurfaces. As shown in Figure 7A–B, growing 60 nm gold nanoparticles on 180 nm-wide reactive regions led to poor particle alignment and disruption of the long-range order. Moreover, an average of 1.6 ± 0.8 particles per region was obtained, highlighting the intrinsic statistical limitation in achieving single-particle growth when the available reactive area is too large. A significant improvement is obtained when moving to smaller reactive regions, which confines the nanoparticles within smaller areas, substantially reducing their misalignment. SEM images reported in Figure 7C–F clearly show a sequential improvement of the gold-particle- patterned substrates by reducing the polymer patch diameter (Ø) from 180 to 120 nm and finally to 70 nm (statistical analysis can be found in the Supporting Information, S13). In addition to improved alignment, control over the number of particles per region also increases, reaching an optimal average of 1.0 ± 0.6 particles per reactive area, much closer to the ideal 1 particle per region.

**Figure 7:**
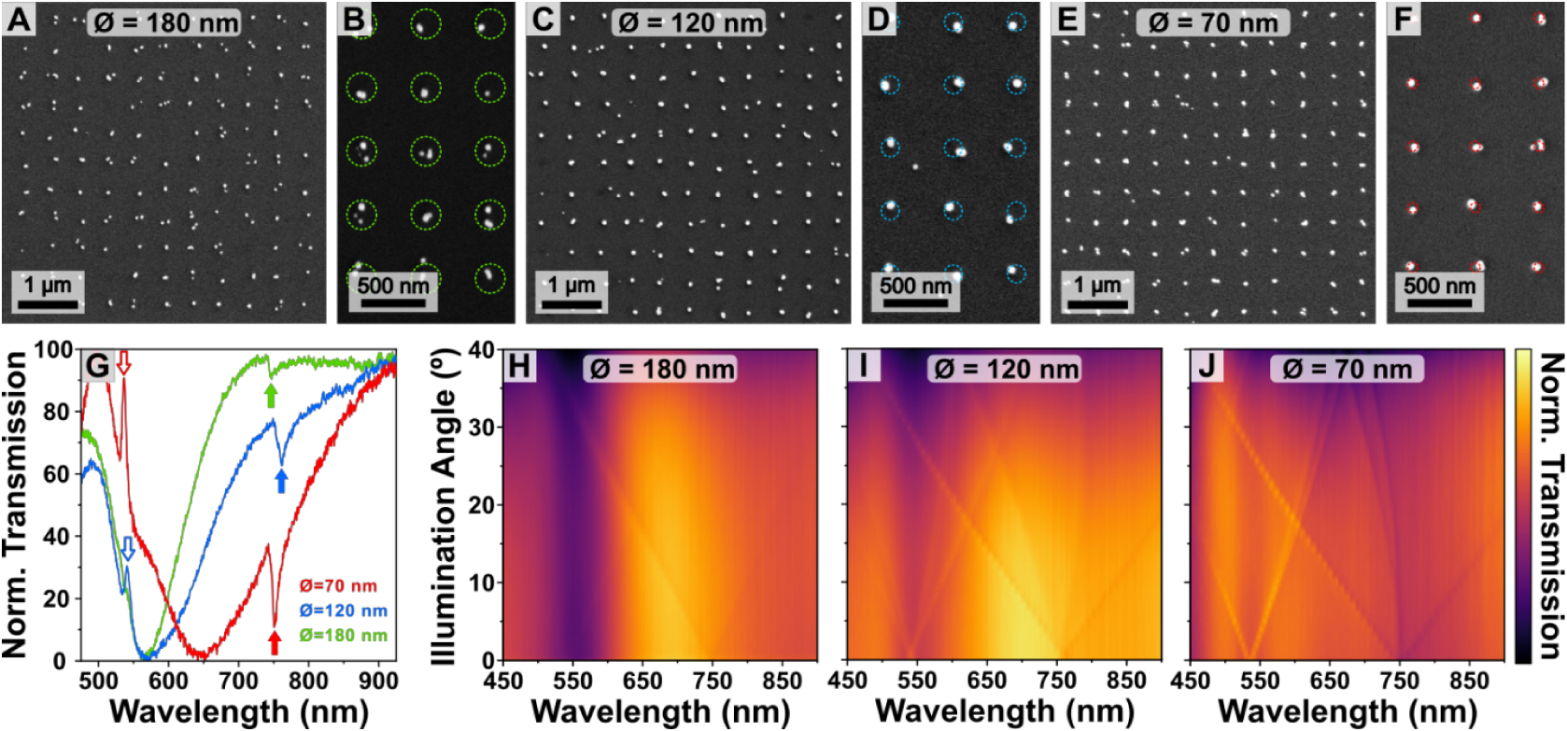
Effect of the patch dimension on the optical properties of *in situ* grown plasmonic metasurfaces. **A–F:** SEM images of gold plasmonic arrays grown on glass substrates with Λ = 500 nm and a patch diameter (Ø) of 180 (**A, B**), 120 (**C, D**), and 70 nm (**E, F**). The colored dotted rings indicate the patch dimension. **G**: Normalized transmission spectra at normal incidence for Ø = 180 (**green**), 120 (**blue**), and 70 nm (**red**). **H–J:** Contour plots of the angular dispersion behavior between normal incidence and θ = 40° for Ø = 180 (**H**), 120 (**I**), and 70 nm (**J**). The same plots with the theoretical line overlays can be found in Supporting Information, S15.

The optical response of the fabricated metasurfaces was first characterized by transmission spectroscopy. Prior to spectral acquisition, an SU8 layer was spin-coated on top of the gold-patterned substrates to ensure a more uniform refractive index environment around the plasmonic array, promoting the far-field coupling between the localized plasmons of the patterned gold nanoparticles and leading to the emergence of collective lattice plasmonic resonances.^16^ The spectral position of the collective lattice modes can be predicted by theory (Supporting Information, S15) using the following simplified equation (valid for square arrays):^49^

1 $$\lambda \left(nm\right)=\frac{\Lambda_{x,y}}{q}\left(n\pm \sin\theta \right),\;\mathrm{with}\;q=\sqrt{m^{2}+l^{2}}$$

Where *Λ*_*x,y*_ represents the lattice parameters in the two orthogonal in-plane directions, *n* is the refractive index of the surrounding medium, *θ* is the illumination incidence angle, and *l* and *m* are signed integers referring to diffraction orders. Assuming an effective refractive index of 1.52, a normal illumination incidence (*θ* = 0°), and a *Λ*_*x=y*_ of 500 nm, eq 1 predicts the emergence of the first- (*q* = 1) and second-order $\left(q=\sqrt{2}\right)$ diffraction lines at 760 and 537 nm, respectively. Figure 7G compares the normalized transmission spectra at normal incidence of *in situ* grown gold nanoparticle arrays with *Λ* = 500 nm and patch diameters (Ø) of 180 nm, 120 nm, and 70 nm. For the 180 nm-wide patches, the transmission spectrum reveals an almost undetectable lattice plasmon resonance peak close to the predicted wavelength (745 nm, **green arrow** in Figure 7G), while there is no trace of higher diffraction modes. This is likely due to the misalignment resulting from the larger available active surface compared to the small 60 nm grown nanoparticles. When the polymer patch dimension was reduced, a clear narrowing of the SLR optical signature can be observed from the normal incidence for both Ø = 120 nm (at 761 nm, **blue arrow**) and Ø = 70 nm (at 753 nm, **red arrow**) samples. Moreover, a second-order diffraction mode was observed at 540 and 537 nm (**blue** and **red empty arrows** in Figure 7G, respectively). The narrow transmission peak appearing within the broader LSPR-related dip can be interpreted as a Fano-like lattice resonance arising from the coupling between the particle LSPR and the second diffractive order of the array. In this case, the collective mode interferes constructively with the transmitted background, giving rise to a peak-like feature rather than a dip.^50^ These results prove that the reduction of the polymeric active patch area leads to tighter control over nucleation position, translating directly into higher large-scale uniformity of the produced plasmonic metasurfaces. This can be quantified by calculating the quality factor (*Q*_f_) of the detected SLRs as:

2 $$Q_{f}=\frac{\omega }{\Delta \left(\omega \right)}$$

where *ω* is the spectral peak position (nm), and *Δ­(ω)* is the full width at half-maximum of the peak. Besides, for the Λ = 500 nm lattice with 180 nm patche size (Ø), *Q*_f_ could be evaluated for Ø = 120 nm and Ø = 70 nm plasmonic arrays for both first- and second-order modes, resulting respectively in *Q*_f,120–first_ = 55, *Q*_f,120–second_ = 71, *Q*_f,70–first_ = 102, and *Q*_f,70–second_ = 131 (details on *Q*_f_ calculations are reported in Supporting Information, S16). These are among the highest *Q*_f_ values reported to date for single-particle colloidal metasurfaces in the visible range. Finally, the diffractive nature of the collective resonances was evaluated through angular transmission measurements on an automated custom-made optical set up (details reported in Supporting Information, S15) with an angle resolution of 1°. The obtained contour plots (Figure 7H–J) reveal the expected angular dependence behavior, where the lattice plasmon resonances split and shift for increasing values of θ. The generated branches become more evident as the biopolymer patch size (Ø) is reduced, and for the case of Ø = 70 nm, the angular dispersion can be followed up to *θ* = 40°. Also, in this case, we observed excellent agreement with theory (Supporting Information, S16).

## Conclusions

In this work, we introduced a robust and scalable strategy for the patterned *in situ* growth of plasmonic nanoparticle arrays based on the confined polymerization of catecholamine biopolymers. By exploiting the spontaneous self-polymerization of DA and NE under mild aqueous conditions, we generated nanometric PDA and PNE patches that provide a chemically defined and spatially confined platform for selective gold nucleation and growth. This patterning approach enables precise control over the nucleation sites, overcoming major limitations of *in situ* growth strategies related to uncontrolled nucleation, poor reproducibility, and limited scalability. The combination of confined polymerization with a seed-mediated growth protocol allowed the decoupling of nucleation and growth processes, effectively suppressing secondary nucleation in solution while preserving tight control over nanoparticle size, density, and spatial distribution. Importantly, controlling the nanoparticle size below the 20 nm regime is particularly relevant for future extensions toward anisotropic growth, targeting crystal twinning and symmetry breaking.^51^ This aspect is even more exciting in the case of *in situ* growth, as the presence of a substrate could introduce new elements of control over the synthetic mechanism, possibly controlling the orientation of the grown nanoparticles with respect to the surface.

By tuning both the dimensions of the reactive biopolymer patches and the seeding conditions, we achieved ordered plasmonic arrays approaching the single-particle-per-site regime. Importantly, the methodology proved to be highly versatile, being readily transferable across substrates of fundamentally different natures.

From an optical standpoint, the resulting metasurfaces sustain ultranarrow surface lattice resonances, with quality factors exceeding 130—among the highest reported to date for single-particle colloidal metasurfaces. These results demonstrate that nanoscale control over the nucleation area is a critical parameter to minimize particle misalignment and disorder, directly translating into enhanced long-range coupling and improved optical coherence across the array.

Beyond the specific system investigated here, the catecholamine-driven patterned *in situ* growth strategy introduced in this study represents a general and conceptually simple platform for the bottom-up fabrication of plasmonic metasurfaces with minimal instrumentation requirements. From an operational standpoint, the entire procedure (from clean substrate to fully grown metasurface) can be completed within an hour. As such, the mild, aqueous, and environmentally benign nature of the process, combined with its scalability and substrate independence, makes it particularly attractive for applications where conventional lithographic or assembly-based approaches remain impractical.

## Materials and Methods

### Materials

Trichloro­(1H, 1H, 2H, 2H-perfluorooctyl)­silane (97%), dopamine hydrochloride, L-norepinephrine hydrochloride (NE, ≥98.0%), 2-amino-2-(idrossimetil)-1,3-propandiolhydrochloride (Tris-HCL, ≥99.0%), sodium hypochlorite, sodium borohydride, HAuCl_4_·3H_2_O (99%), l-ascorbic acid (99%), hexadecyltrimethylammonium bromide (CTAB, ≥96.0%), hydrochloric acid, sulfuric acid (95–98%), sodium hydroxide, nitric acid, hydrogen peroxide (30%), toluene, hexane, and acetone were purchased from Merck (Darmstadt, Germany). 2-Propanol (IPA, 99.8%), ethanol, and glass slides were purchased from Labbox (Barcelona, Spain). SU-8 2000.5 and SU-8 2000 Thinner were purchased from Kayaku (Westborough, USA). Polydimethylsiloxane (PDMS, Sylgard 184) was purchased from Dow Corning (Michigan, USA). The hard PDMS (hPDMS) mixture components were purchased from Gelest (CymitQuimica, Spain): (7.0–8.0% vinylmethylsiloxane)-dimethylsiloxane copolymer, trimethylsiloxy terminated, 1,3,5,7-tetramethylcyclotetrasiloxane (95%), platinum-divinyltetramethyldisiloxane complex (2% Pt in xylene), and (25–35% methylhydrosiloxane)-dimethylsiloxane copolymer, trimethylsiloxane terminated. OrmoStamp and OrmoPrime were purchased from Micro Resist Technology (Berlin, Germany). All the masters were purchased from ThunderNIL (Trieste, Italy). Ultrapure Milli-Q water (R ≥ 18.2 MΩ cm) was used for the preparation of buffer solutions. All chemicals were used as received.

All glassware and magnetic stir bars used for synthesis were cleaned thoroughly with aqua regia (3:1 concentrated HCl to HNO_3_) and rinsed with Milli-Q water before use. Glass slides were cleaned thoroughly with piranha solution (3:1 95–98% w/w H_2_SO_4_ and 30% H_2_O_2_), rinsed with Milli-Q water, and stored in Milli-Q water prior to use.

### Methods

#### PNE and PDA Patterned Samples Preparation

##### Single Pattern Imprinting

PNE and PDA patterns were obtained on different precleaned substrates (silicon, glass, and silicon-supported flat sPDMS). The polymerization was carried out at room temperature for 10 min by alkali-induced autoxidation of the monomer (NE or DA, 2 mg/mL) in 10 mM Tris-HCl buffer (pH 8.5) under mild pressure (0.5 bar). A 2 μL droplet of the monomer solution was drop-cast onto a cleaned substrate and immediately covered with the template sPDMS stamp (Supporting Information, S2), carefully positioned to avoid lateral movements. The sandwich assembly was then pressed using a custom-made press, reaching a constant pressure of 0.5 bar. After 10 min, the pressure was released, the sPDMS template was gently demolded, and the patterned substrate was rinsed with Milli-Q water for ∼1 min before drying with nitrogen. For large-scale features (spiral and line), the polymerization time was extended up to 60 min to ensure complete and homogeneous pattern replication. In addition, hPDMS was employed to minimize template deformation and collapse effects during polymerization.

After each use, the patterned sPDMS or hPDMS templates were cleaned by sequential sonication in sodium hypochlorite (5 min), Milli-Q water (5 min), and isopropanol (5 min), followed by drying in an oven at 60 °C for at least 15 min. After cleaning and before their reuse, s/hPDMS stamps were left to cool down at room temperature. Notably, this cleaning procedure allows a single stamp to be reused for over 30 experimental cycles without loss of pattern integrity. Detailed procedures and reaction conditions for the PNE and PDA-patterned polymerization are reported in Supporting Information (S2.1 and S2.2).

##### Large-Scale and Multi-Pattern Imprinting

This procedure is easily scalable to larger substrates. By employing sPDMS or hPDMS templates containing larger or multiple patterns, samples up to 5 × 5 cm^2^ with 9 different patterns were fabricated in this work. For each patterned region, a 2 μL droplet of monomer solution was deposited, with the applied pressure adjusted according to the pattern size. See Supporting Information, S2.6 and S2.7 for more details.

#### *In Situ* Gold Nanoparticle Growth

Gold nanoparticle arrays were obtained using a two-step seed-growth procedure, in which nucleation occurred directly on the PNE/PDA substrates, followed by a growth step.

##### Seeding

Approximately 300 μL (sufficient to cover the entire substrate) of an aqueous HAuCl_4_ solution (0.02 mM) was drop-cast onto the PNE/PDA substrates and allowed to react for 5 min. Samples were then rinsed with Milli-Q water and dried with nitrogen. The adsorbed Au^3+^ was subsequently reduced by adding 300 μL of an aqueous NaBH_4_ solution (0.6 mM), which was drop-cast and allowed to react for 10 min. After reduction, the substrates were rinsed with Milli-Q water, kept under a drop of water for 30 min, rinsed again, and finally dried with nitrogen. The step with water was introduced to deactivate any possible residuals of NaBH_4_ might be left on the biopolymer-patterned substrates.

##### Growth

The growth step was carried out following an established *in situ* procedure.^25^ Briefly, 12 μL of a 50 mM aqueous HAuCl_4_ solution was added to 1 mL of 25 mM CTAB (capping ligand/surfactant) and sonicated. Subsequently, 12 μL of a 0.1 M aqueous solution of ascorbic acid was added under rapid stirring. Within approximately 5 s, the solution rapidly changed color, fading from orange to transparent. Immediately after this transition, a 150 μL aliquot of the growth solution was drop-cast onto the substrates and left to react for 1 min. The substrates were then rinsed thoroughly with Milli-Q water and dried with nitrogen.

Detailed procedures for the *in situ* growth of gold nanoparticles, and their density and dimension tuning, are reported in Supporting Information (S7, S8, and S9).

#### Characterization

##### Scanning Electron Microscopy (SEM)

SEM analyses were performed using a Phenom Pharos G2 desktop field emission gun scanning electron microscope (FEG-SEM) (Thermo Fisher Scientific, Waltham, MA, USA). Images on silicon wafers were acquired under high-vacuum conditions (0.10 Pa) with an acceleration voltage of 10 kV, employing the secondary electron detector (SED) without any metal vapor deposition pretreatment prior to image acquisition. Images on glass slides, supported sPDMS and hPDMS, were acquired under low-vacuum conditions (60 Pa) with an acceleration voltage of 5 kV, employing the backscattered electron detector (BSD).

##### Atomic Force Microscopy (AFM)

AFM measurements were carried out using an ezAFM system (NanoMagnetics Instruments) under ambient conditions, mounted on a home-built antivibration platform. The instrument was operated in dynamic (tapping) mode using cantilevers with a nominal spring constant of 58 N/m and a resonance frequency of approximately 190 kHz. The analyses were performed using WSxM software,^52^ scanning squared areas of 10 × 10 μm^2^ and 5 × 5 μm^2^ with scan rates of 10 μm/min and 5 μm/min, respectively. Before proceeding with height profile analyses, topography images were first treated with a flatten filter to remove low-frequency noise and finally with an equalize filter to better enhance the contrast. The same corrections were applied to the phase-contrast AFM.

##### Angular Displacement with Optical Measurements

Prior to spectra acquisition, a thin SU-8 layer (1:1 SU-8 2000.5/SU-8 2000 Thinner) was spin-coated (500 rpm, 100 rpm/s, 10 s) on top of the gold-patterned substrates to ensure a more uniform refractive index environment around the plasmonic array. The experimental optical setup was customized using LabVIEW-licensed programs to automatize the measurements. The plasmonic patterned area was illuminated at normal incidence using white light produced by a Tungsten Halogen Lamp (Ocean Optics, HL-2000-HP, Florida, USA). The light was collimated on the sample and later collected using two achromatic doublet lenses (f = 50.00 mm and f = 30.00 mm). The sample holder, positioned between the two achromatic doublet lenses, was anchored on a rotational stage (*Ø* = 50 mm) with resonant piezoelectric motors (Thorlabs, ELL18/M) equipped with an interface board controlled by software. This allowed the automatic rotation of the illumination angle θ (from 0° to 40°) of the sample, with a resolution of 0.1°. Samples were mounted vertically and oriented along the high-symmetry direction of the array on a custom-made sample holder, which enabled control of the azimuthal angle φ (±3°) alignment. The transmitted light was collected using a fiber-coupled spectrophotometer (Ocean Optics, Mayan2000) with a detection range of 380–1200 nm.

## Supplementary Material



## Notes

Experimental details and tips for master replica; PDMS stamp preparation; optimization of all the parameters for confined polymerization and nanoparticle growth; all data on PDA patterning; control experiments for patterned *in situ* growth; statistical analysis of particle density, particle size, and patterning defects; additional SEM characterization; details on custom-built optical setup and optical measurements; and details on quality factor calculation (PDF)
