# Supplementary material for: One-Step Confined Polymerization of Catecholamine Biopolymers for the Patterned In Situ Growth of Plasmonic Metasurfaces with Single-Particle Resolution
Source: ACS Appl Mater Interfaces. 2026 Jun 1;18(24):34013–23. doi: 10.1021/acsami.6c05739 (PMC13307077; doi:10.1021/acsami.6c05739)
Supplement: Supplementary file 1 [file am6c05739_si_001.pdf]

Supporting Information

Title: One-Step Confined Polymerization of Catecholamine Biopolymers for the Patterned *in Situ* Growth of Plasmonic Metasurfaces with Single Particle Resolution

Serena Schiavi[a†], Simone Ventisette[b†], Pau Vilches Rueda[c], Maria Minunni[d], César Moreno[e], Angelo Taglietti[a], Simona Scarano[b], Leonardo Scarabelli[c*]

[a] Department of Chemistry, University of Pavia, 27100 Pavia, Italy

[b] Department of Chemistry “Ugo Schiff”, University of Florence, 50019 Sesto Fiorentino, Italy

[c] NanoOddLAB, Department of Chemistry and Process & Resource Engineering, ETSIIT, University of Cantabria, 39005 Santander, Spain

[d] Department of Pharmacy, University of Pisa, 56126 Pisa, Italy

[e] Departamento de Ciencias de la Tierra y Fisica de la Materia Condensada, Universidad de Cantabria, 39005 Santander, Spain

[†] S. Schiavi and S. Ventisette contributed equally.

CONTENTS

S1. Soft lithography: replica of the original master and fabrication of the soft PDMS (sPDMS) stamp

S1.1 Preparation of the original master replica with Ormostamp

S1.2 Preparation of hPDMS holes stamps

S1.3 Preparation of operative masters with SU8 2000.5

S1.4 Preparation of sPDMS stamps

S2 Optimization of confined polymerization protocol

S2.1 Substrate preparation

S2.2 Experimental details for the confined polymerization procedure

S2.3 sPDMS/hPDMS template cleaning procedure

S2.4 Polymerization time optimization

S2.5 Pressure optimization

S2.6 Protocol variation for micron scale arrays

S2.7 Scale-up of the confined polymerization for simultaneous multi-patterning

S3. Analysis of polymer contraction after polymerization

S4. SEM images of PDA patterned substrates on silicon

S5. *In situ* growth control experiments

S6. AFM images analysis on PNE patterns after the seeding step

S7. Experimental details *in situ* growth conditions

S8. Analysis of particle density

S9. Analysis of particle size

S10. SEM images PDA *in situ* growth

S11. Additional SEM characterization

S12. *In situ* growth on PDMS

S13. Statistical analysis of particle density by reducing polymer patches area

S14. Reproducibility inter-sample

S15. Details on optical set up and optical measurements

S16. Details on Q_f calculation

S1. Soft lithography: replica of the original master and fabrication of soft PDMS (sPDMS) stamp

One of the advantages of nanoimprint lithography is the possibility of fabricating a virtually infinite number of PDMS stamps from a single original master. To do so, exact replicas called *operative masters* are prepared, from which the desired PDMS stamp can be produced (tens of PDMS preparations for each generation of *operative masters*). The original silicon masters of square, hexagonal lattice and linear gratings are made of cylindrical holes with the parameters listed in **Table S1**. The original silicon masters of square lattice with 500 nm of lattice period and reduced features and the spiral are made of pillars with the parameters listed in **Table S1**. All the masters were purchased from ThunderNIL (Trieste, Italy).

Table S1. Geometrical parameters of silicon masters.

Lattice period (Λ)	Feature diameter
400 nm (squared)	230 nm (Holes)
500 nm (squared)	277 nm (Holes)
600 nm (squared)	338 nm (Holes)
600 nm (hexagonal)	295 nm (Holes)
600 nm (linear grating)	299 nm (Holes)
500 nm (squared)	150 nm (Pillars)
500 nm (squared)	100 nm (Pillars)
12 μm Spiral	8×7 μm (Pillars)

First, hard PDMS (hPDMS) stamps are prepared directly from the original masters. These are then used to prepare several *operative masters* using thermal nanoimprint lithography on SU8 layers. For silicon masters consisting of hole arrays, an additional step is needed in order to generate an *operative master* consisting of a complementary pillars array. Specifically, the master is first replicated using Ormostamp, obtaining a hard complementary replica of the original pattern. From this, standard hPDMS/SU8 combination is used to fabricate the desired *operative masters* and sPDMS stamps. The

following sections report the experimental details of master and stamp preparation. **Figure S1** shows a brief scheme of the process to fabricate the sPDMS stamps.

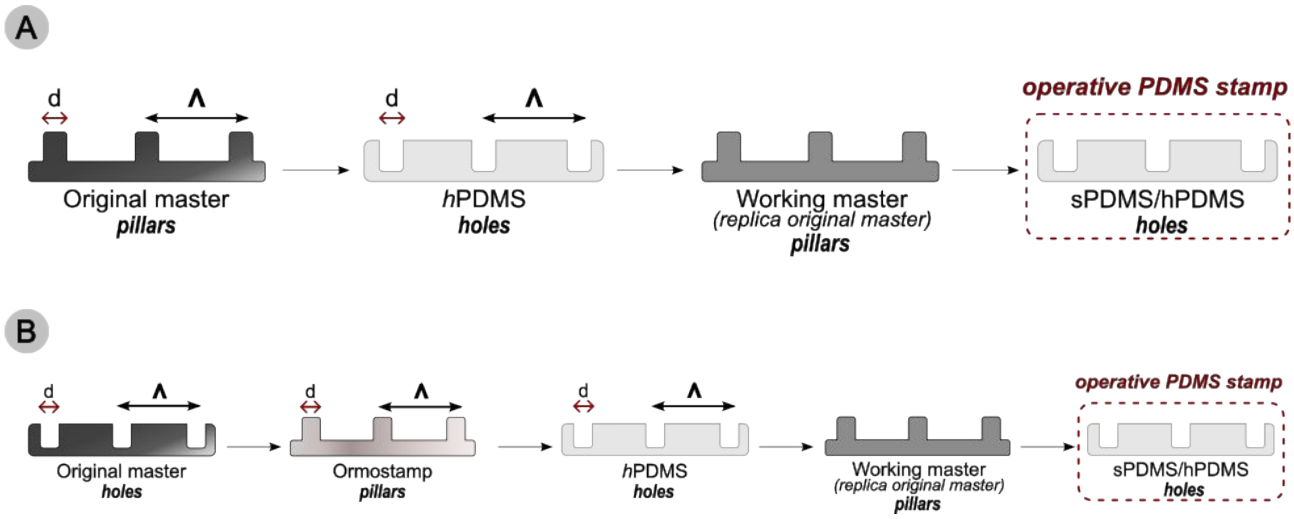

Figure S1. Schematic of the fabrication process to produce sPDMS stamp from (**A**) original master of pillars and (**B**) from original master of holes.

S1.1 Preparation of the original master replica with Ormostamp

The holes array silicon masters were first treated by silanization *via* chemical vapor deposition of perfluorooctyl-trichlorosilane, under vacuum in a desiccator for 5 min. This treatment protects the masters and avoids the Ormostamp sticking permanently to the silicon surface. Once the deposition was completed, the masters were rinsed with acetone and heated at 120 °C for 20 min in order to remove all silane leftovers. Afterwards, a drop of Ormostamp photoresist was placed directly on top of the silanized silicon master. A cleaned glass slide, first spin-coated with Ormoprime (4000 rpm, 1000 rpm/s, 60 s), was gently superimposed on the master, making sure that no bubbles remain trapped between the glass and the Ormostamp layer. The photoresist was then activated by exposure to 1 Joule of direct UV light. Finally, cross-linking was favored by placing the substrates on a hot plate and applying a temperature ramp from 60 to 150 °C over the course of 30 min. Apart from the curing of the photoresist, the difference of thermal expansion coefficient between the photoresist and silicon induces the Ormostamp to easily detach from the silicon master, yielding an Ormostamp master of pillars arrays, which can be used for the preparation of hPDMS stamps.

S1.2 Preparation of hPDMS holes stamps

The Ormostamp masters (or the original silicon masters in the case of pillar arrays) were replicated using hPDMS. This prevents the collapse of the structure during the next nanoimprint lithography step. The hPDMS composite was applied by drop-casting on the master of choice, yielding a thickness of a few microns, while the backbone of the replica was composed of sPDMS to ensure flexibility. Specifically, the hPDMs mixture was prepared by adding under vigorous stirring and in sequence: 1.7 g vinylmethylsiloxane, 50 µL of 1,3,5,7-tetracetylcyclosilane, 4 µL of Pt catalyst, 550 µL of hydroxyl siloxane, and 2 mL of toluene. The sequence of addition should be performed as fast as possible, since the hPDMS mixture will immediately start to cure. Due to toluene quick evaporation, the obtained mixture can be used for approximately 30 min, before solidification takes place. The mixture was then drop-casted on top of the patterned area and spread over the entire surface by using a compressed air gun, ensuring to not leave air bubbles trapped in the layer over the patterned area. This process was repeated at least 3 times for each sample to ensure complete coverage. The substrates were left for 1 h at room temperature and then another hour on a hot plate at 60 °C to ensure complete evaporation of toluene. Afterwards, sPDMS was prepared by mixing the monomer and curing agent in 10:1 ratio, degassing the mixture by centrifugation at 3500 rpm for 5 min and casting it onto a Petri dish containing the cured hPDMS-coated masters. After complete air bubble removal by degassing under vacuum, the composite hPDMS-sPDMS stamps were cured at 60 °C for at least 4 h prior to carefully demolding them from the masters. The obtained hPDMS hole arrays are ready and were further employed for the preparation of the operative masters.

N.B.: Due to their small features, for the L500-d140 and L500-d80 masters the hPDMS mixture was directly cast and spread with an air gun at 65 °C on a hotplate and cured for 1 h at the same temperature.

S1.3 Preparation of operative masters with SU8 2000.5

First, the hole array hPDMS stamps prepared as previously described were functionalized by vapor-phase deposition of perfluorooctyltrichlorosilane and cleaned with isopropanol. The epoxy-based negative photoresist SU8 2000.5 was then spin coated following a two-steps procedure (first step: 500 rpm, 100 rpm/s, 10 s; second step: 2000 rpm, 300 rpm/s, 30 s) on cleaned glass or silica wafer and then placed on a hot plate. The temperature was increased to 120 °C and the hPDMS stamp was gently superimposed and slowly pressed into the SU8 layer using a finger or a tweezer. Rapidly, the stamp deformed the epoxy layer. The obtained substrate-stamp assembly was left to cool down at room temperature, after which the hPDMS stamp could be carefully demolded, leaving a nanopatterned area on the epoxy-based photoresist. Finally, the SU8 layers were exposed to direct UV-light (80 mJ) and post-baked for 60 min on a hot plate at 150 °C.

S1.4 Preparation of sPDMS stamps

sPDMS templates were used for the PNE/PDA confined polymerization, resulting in flexible and durable stamps even after several uses (over 30 preparations). The polymer mixture was prepared by combining a 10:1 ratio of monomer and curing agent in falcon tubes of 50 mL weighting around a total of 35 g. The two components were vigorously mixed for several minutes, to ensure their homogeneous distribution and subsequently, to remove air bubbles, the tubes were centrifuged at 3500 rpm for 5 min. The mixture was then cast onto the petri dishes containing the operative SU8 masters, degassed under vacuum and cured at 60 °C for at least 4 h. Finally, the sPDMS stamps were carefully demolded from the operative masters.

Prior to use, the sPDMS templates were treated with a deep washing cycle in an ultrasonic bath to ensure the complete removal of any unreacted residual oligomers that could affect the quality of the resulting plasmonic metasurfaces. The cycle consists in the ultrasonication of the freshly obtained sPDMS stamp first in ethanol for 5 min, followed by ultrasonication in hexane for 30 min and drying in an oven at 60 °C for 30 min. The cycle was repeated three times, keeping the clean sPDMS stamp in the oven for 60 min after the last cycle and letting it cool down to room temperature before use.

S2. Optimization of confined polymerization protocol

S2.1 Substrate preparation

- Silicon wafers

Silicon wafers were cut into square pieces (1×1 cm^2), sonicated in isopropanol for 5 min, and dried with compressed air prior to use.

- Glass slides

Glass slides were cut into square pieces (1×1 cm^2), cleaned thoroughly with piranha solution, rinsed with Milli-Q water, dried with nitrogen, and stored in Milli-Q water until use.

- Silicon-supported flat sPDMS

Silicon-supported flat sPDMS substrates were prepared using ~ 3 mL of PDMS 10:1 ratio mixture combined with ~ 7 mL of toluene. The mixture was vigorously stirred to ensure homogeneity, centrifuged at 3500 rpm for 5 min to remove air bubbles, and further degassed under vacuum for 15 min. A drop of the resulting PDMS mixture was then deposited onto a cleaned silicon and spin-coated into a thin film of ~ 2 microns (5000 rpm, 1500 rpm/s, 60 s). The coated substrates were cured in an oven at 60 °C overnight. Prior to use, the substrates were activated by UV-ozone treatment (Jelight Model 42) for 15 min to promote surface hydrophilicity.

S2.2 Experimental details for the confined polymerization procedure

The confined polymerization of NE and DA was carried out using the custom-made press shown in **Figure S2**. The device consists of a motorized press equipped with electronics controlling pressing speed and height, operated *via* a custom-made controller. Operatively, a clean 1×1 cm^2 slice of silicon (or glass/silicon-supported flat sPDMS) is placed on a digital kitchen scale positioned directly beneath the press. In the meantime, ~ 5 mL of Tris-HCl buffer (pH 8.5) are placed in a vial. While stirring, NE or DA powder is added to the Tris-HCl solution, reaching a final monomer concentration of 2 mg/mL. As soon as the monomer is completely solubilized (in about 30 s), 2 μL of the solution is drop-cast in the middle of the silicon substrate and immediately covered with the patterned PDMS stamp. The motorized press is then used to apply constant pressure on the obtained sandwich, using the scale to stop the motor at the desired load. After 10 min, the pressure is released by lifting the

moving press, the PDMS template was gently demolded, and the patterned substrate was rinsed with Milli-Q water for ~ 1 min before drying with nitrogen.

All the experiments carried out to assess the optimal polymerization parameters and to extend the protocol to all the arrays used in this work were carried out using this first press prototype.

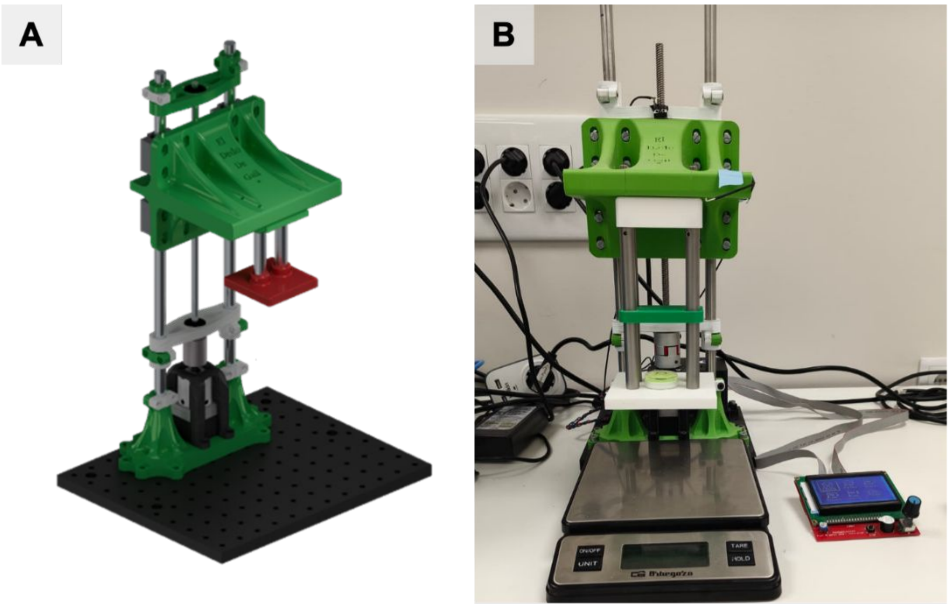

Figure S2. (**A**) Rendering and (**B**) photograph of the motorized press prototype employed for the optimization of the confined biopolymer polymerization protocol. The piece applying the pressure is highlighted in red.

S2.3 sPDMS/hPDMS template cleaning procedure

After each use, the patterned PDMS stamps were cleaned by sequential sonication in sodium hypochlorite (5 min), Milli-Q water (5 min), and isopropanol (5 min), followed by drying in an oven at 60 °C for at least 15 min. After cleaning and before their reuse, PDMS stamps were left to cool down at room temperature.

S2.4 Polymerization time optimization

To further assess the polymerization kinetics under our configuration, we performed AFM and SEM analysis on PNE-patterned substrates obtained after shorter polymerization times (10 and 30 min). **Figure S3** reports AFM phase-contrast images (**A-B**) and topography profiles (**C-D**), together with SEM images (**E-F**), for patterns generated after 10 min (**A, C, E**) and 30 min (**B, D, F**) of confined polymerization under a PDMS stamp.

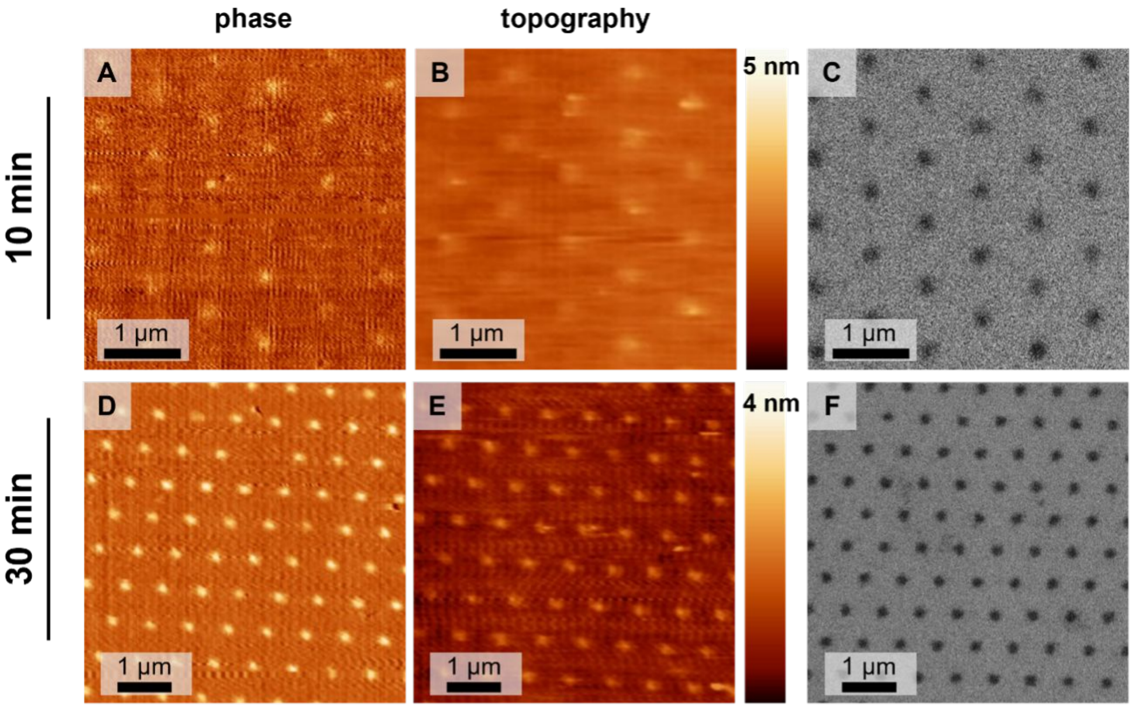

Figure S3. AFM phase-contrast (**A, D**) and topography (**B, D**) images and SEM analysis (**C, F**) performed on PNE squared patterns (Λ = 500 nm) obtained after 10 min (**top row**) and 30 min (**bottom row**) of polymerization.

Analysis of the AFM topography profiles revealed average PNE thicknesses of 0.7 ± 0.2 nm and 0.9 ± 0.2 nm after 10 and 30 min of polymerization, respectively. **Table S2** summarizes the mean values for all polymerization times investigated.

Table S2. PNE thicknesses obtained from topography AFM characterizations after different polymerization times. Each reported value is averaged from 100 different polymer patches.

**Polymerization time**	**Average PNE thickness (nm)**
10 min	0.7 ± 0.2
30 min	0.9 ± 0.2
60 min	0.8 ± 0.2

These analysis confirm that longer polymerization times do not significantly affect the final thickness of the polymer films, supporting our hypothesis that the reaction is self-limiting due to hindered oxygen diffusion through the PDMS stamp, which confines the monomer solution.

S2.5 Pressure optimization

Once the polymerization time was set, the pressure applied was optimized with the goal of ensuring high-fidelity pattern replica. Experiments were performed by fixing the polymerization time at 10 min while varying the applied pressure (0.3, 0.5, and 1 bar) on a hexagonal PDMS stamp array. **Figure S4** shows the corresponding SEM images for each condition.

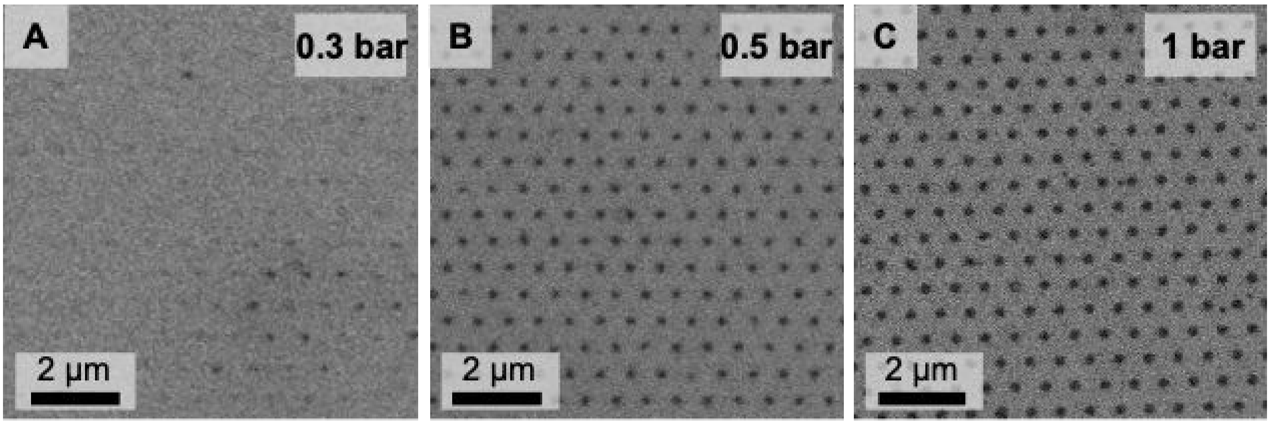

Figure S4. SEM images of hexagonal PNE patterns obtained with 10 min of polymerization applying respectively 0.3 (**A**), 0.5 (**B**) and 1 bar (**C**) of pressure.

As can be observed, when the applied pressure is lowered below 0.5 bar, the patterning procedure fails to yield a homogeneous, extended pattern, likely due to leakage of the monomer solution outside the PDMS holes, resulting in uneven patch formation across the substrate. Accordingly, an applied pressure of 0.5 bar is selected as the optimal condition to ensure uniform, extended pattern formation.

S2.6 Protocol variation for micron scale arrays

For the extension of the confined polymerization protocol to large-area patterns (line and spiral micron-scale arrays), specific adjustments were required. In particular, hPDMS templates were preferentially employed instead of sPDMS, as line and spiral geometries require enhanced mechanical stability to prevent elastic deformation, feature collapse, or local distortions during contact with the substrate under pressure.

Post-polymerization cleaning procedures were adapted to hPDMS stamps, excluding hexane-based treatments, which were found to induce mechanical damage. Instead, a sequential sonication protocol was implemented, consisting of sodium hypochlorite solution (5 min), Milli-Q water (5 min), and isopropanol (5 min), followed by drying in an oven at 60 °C for at least 15 min.

Moreover, the confined polymerization of NE and DA was extended to 1 h instead of 10 min, in order to allow complete polymer formation within the cavities, which would otherwise remain partially unfilled due to the increased feature size and continuity of the patterns (**Figure S5**).

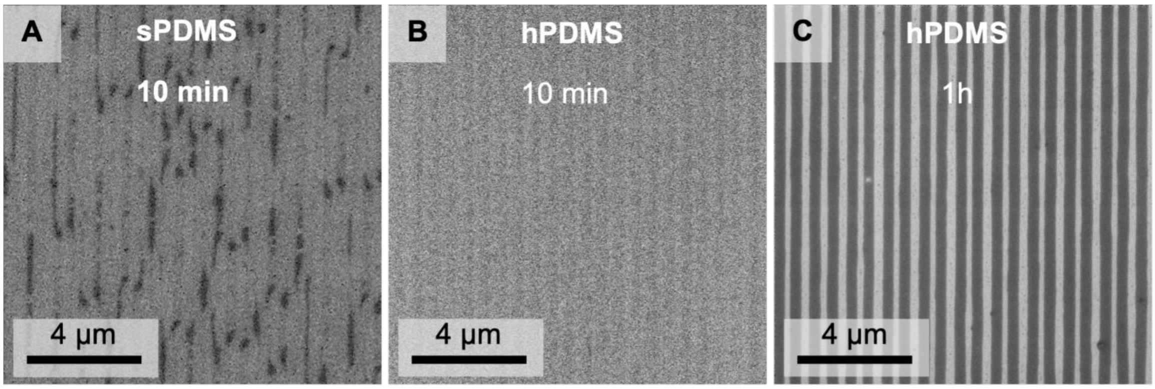

Figure S5. SEM images of line PNE patterns obtained with (**A**) sPDMS - 10 min of polymerization; (**B**) hPDMS - 10 min of polymerization; (**C**) hPDMS - 1 h of polymerization.

S2.7 Scale-up of the confined polymerization for simultaneous multi-patterning

Scaled nanoimprint lithography for large-area master fabrication

To scale up the confined polymerization process and enable the fabrication of large-area PNE/PDA patterns with multiple geometries, the nanoimprint lithography protocol previously described (see **Section S1.3**) was slightly modified in order to place 9 different patterns on a single 5×5 cm^2 silicon substrate.

Briefly, SU-8 2000.5 was spin-coated following the same two-step procedure described previously and brought to 120 °C, crossing the glass temperature transition and softening the epoxy-based resin. An hPDMS stamp was then placed and gently pressed into the SU-8 layer, allowing local deformation of the photoresist and pattern transfer. After cooling to room temperature, the stamp was carefully demolded, leaving a discrete nanopatterned region on the SU-8 layer. After demolding, the imprinted area was selectively exposed to UV light (80 mJ) through a custom-made photomask that prevents undesired crosslinking of non-patterned regions, followed by a post-exposure bake at 150 °C for 60 min. Once the post-bake is completed, the whole softening-imprinting-exposure-demolding-curing-bake cycle was repeated for each geometry (here 9), spread across an area of approximately 5×5 cm^2 (**Figure S6**).

Once the operative large-scale SU-8 master was fabricated, sPDMS/hPDMS composite stamps were prepared as indicated previously (see **Section S1.2** and **Section S1.4**).

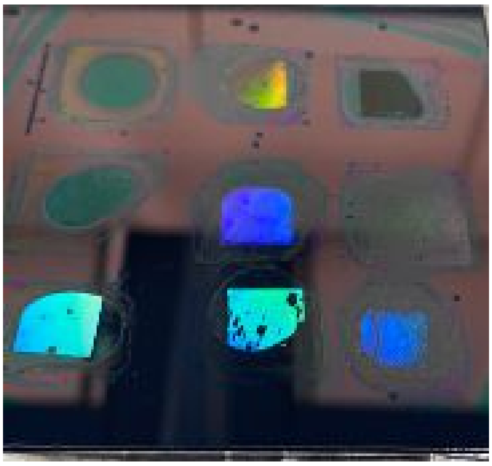

Figure S6. Photograph of patterned SU8 with an array of different periodicity (squared patterns of 400 nm (Λ), 500 nm (Λ) and 600 nm (Λ), hexagonal pattern, spiral pattern, line pattern and squared patterns with periodicity of 500 nm (Λ) with dimension (Ø) of 140 and 80 nm) obtained by thermal nanoimprint lithography.

Scale-up of the confined polymerization protocol

The confined polymerization of NE and DA on large-area substrates and multiple patterned regions was performed using a second custom-made pressing device, shown in **Figure S7**. The setup consists of a manually operated hydraulic press equipped with an integrated pressure sensor, allowing control of the applied load.

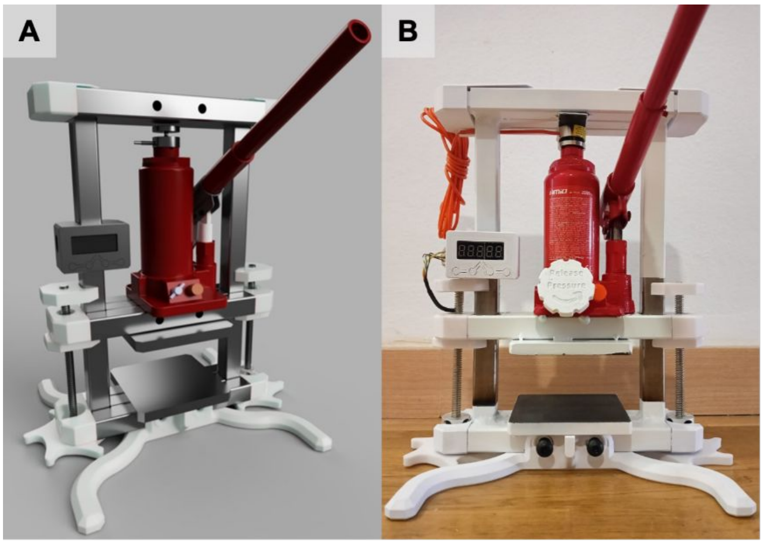

Figure S7. (**A**) Rendering and (**B**) photograph of the motorized press prototype employed for the large-scale polymerization protocol.

Operatively, a clean PDMS composite stamp was placed on the press holder. In parallel, approximately 5 mL of Tris-HCl buffer (pH 8.5) were introduced into a vial. While stirring, NE or DA powder is added to the Tris-HCl solution, reaching a final monomer concentration of 2 mg/mL Upon complete dissolution of the monomer (typically within ~ 30 s), 2 µL aliquots of the freshly prepared solution were rapidly dispensed onto each patterned region of the PDMS composite stamp. The stamp was then immediately covered with a clean 5×5 cm^2 silicon wafer. Subsequently, a constant pressure of 0.5 bar was applied using the hydraulic press, with the applied load carefully monitored and fixed at the desired value *via* the pressure sensor. For a total area of 25 cm^2, a total weight of 12.5 kg was applied.

After 10 min/60 min, the PDMS template was gently demolded, and the patterned substrate was rinsed with Milli-Q water for ~ 1 min before drying with nitrogen.

S3. Analysis of polymer contraction after polymerization

The biopolymer circular patches obtained with the confined polymerization protocol exhibited slightly smaller diameters than those of the corresponding soft and hard PDMS stamp cavities employed. **Figures S8**, **S9**, and **S10** show SEM images of PNE square-lattice patterns on silicon obtained using the same lattice spacing (Λ = 500 nm) but different feature diameters, together with the corresponding SEM images of the PDMS-hole stamps used for the confined patterning. SEM analysis clearly confirms a contraction of the biopolymer circular patch compared with the diameter of the PDMS hole cavity. In **Table S3**, all dimensional parameters are summarized, comparing the PDMS holes diameters measured from SEM and the corresponding PNE patterns for all the squared arrays employed in this work.

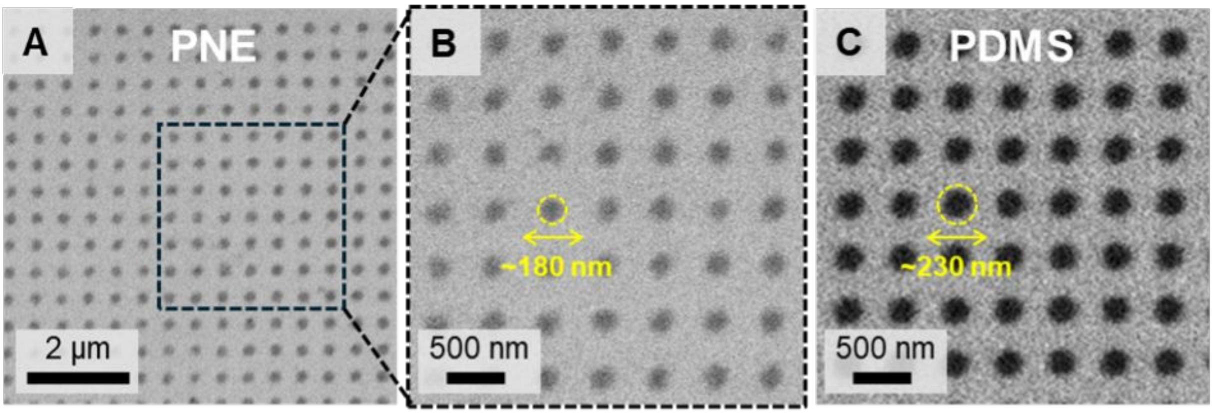

Figure S8. (**A-B**) Representative SEM image of a squared PNE-patterned substrate (Λ = 500 nm) and magnified view highlighting the biopolymer patch diameter. The patch diameter is reduced compared with the corresponding hole diameter of the PDMS stamp used for confined patterning (**C**).

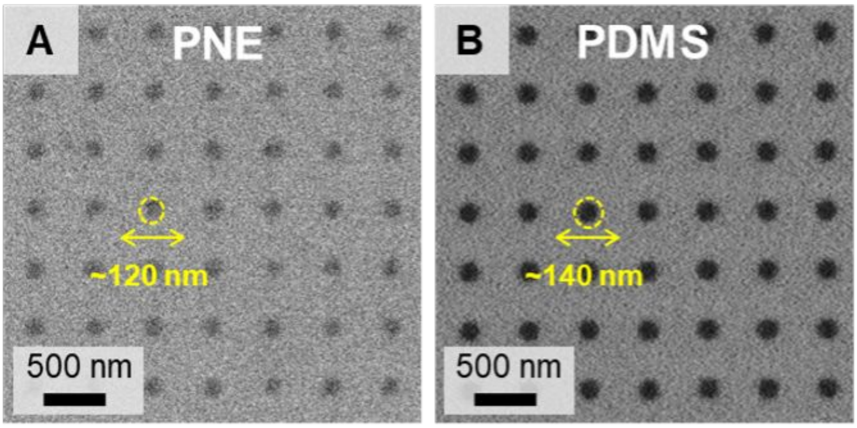

Figure S9. Representative SEM image of PNE patterns with Λ = 500 nm (**A**) and of PDMS holes stamp (**B**) employed during the polymerization process.

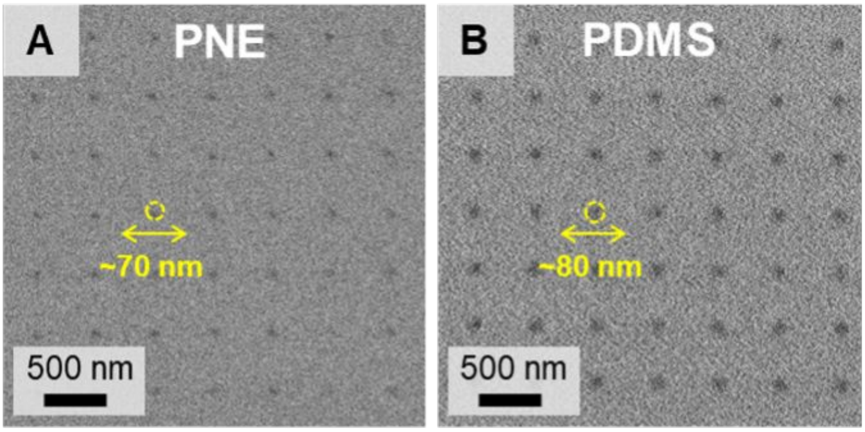

Figure S10. Representative SEM image of PNE patterns with Λ = 500 nm (**A**) and of PDMS holes stamp (**B**) employed during the polymerization process.

Table S3. Summarized dimensional analysis obtained from SEM images of the PDMS holes and the corresponding PNE-patterned substrates. The reported dimensions were calculated by averaging > 100 diameters per sample. † Values reported in the main text used to distinguish among the different 500 nm square-lattice arrays.

Lattice array	PDMS holes cavities diameter (nm) †	PNE patterns diameters (nm)
400 nm	190 ± 10	170 ± 10
500 nm	230 ± 10	180 ± 10
500 nm	140 ± 10	120 ± 10
500 nm	80 ± 10	70 ± 10
Hexagonal (600 nm)	230 ± 10	200 ± 10
600 nm	260 ± 10	210 ± 10

S4. SEM images of PDA patterned substrates on silicon

The confined polymerization protocol, described in the manuscript for PNE, was also successfully employed to obtain PDA patterned patches of different geometries and lattices. **Figure S11** reports SEM images acquired for each PDA patterned substrate on silicon.

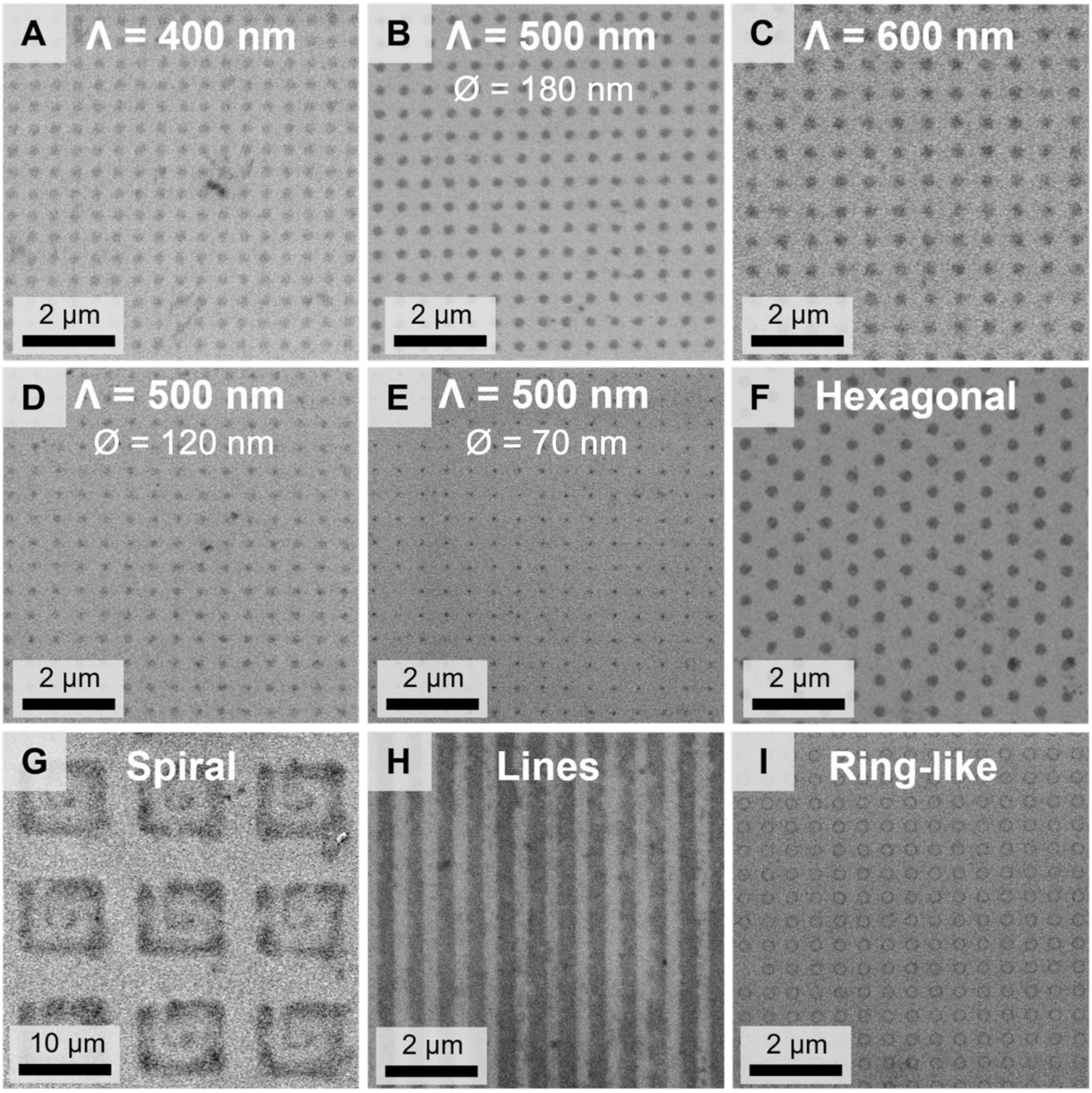

Figure S11. SEM images of PDA patterned substrates after confined self-polymerization of DA under PDMS stamps with different geometries and lattices, yielding to: (**A-C**) squared lattices with periodicities (Λ) of respectively 400, 500 and 600 nm; (**D, E**) squared lattices of Λ = 500 nm and reduced dimensions of polymer patches with calculated values of respectively 120 and 70 nm; (**F**) hexagonal lattice; (**G**) spiral; (**H**) line pattern and (**I**) squared ring-like pattern with lattice period of 500 nm.

S5. Negative control experiments *in situ* growth

To ensure full control over each stage of the fabrication process, and to confirm the hypothesized growth mechanism, a comprehensive set of control experiments was performed by selectively omitting individual steps of the optimized single-particle growth protocol. The reference conditions were those optimized for single-particle formation: PNE and PDA confined polymerization was performed with a pressure of 0.5 bar and 10 min under 0.5 bar pressure; gold seeding with 0.02 mM Au^{3+} for 5 min followed by reduction with 0.6 mM $NaBH_4$ for 10 min; and a final *in situ* growth step of 1 min**.**

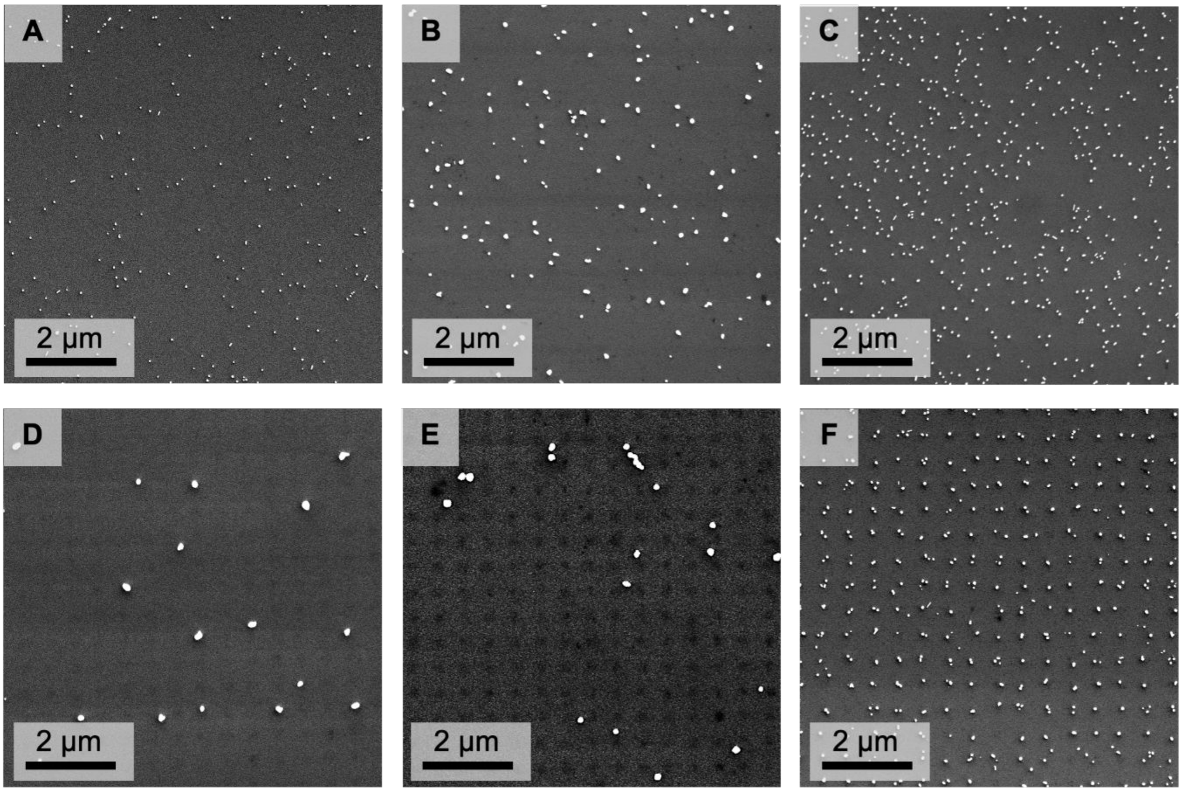

Figure S12. Control experiments illustrating the role of each step in the *in situ* gold nanoparticle growth on PNE/PDA substrates. (**A**) Absence of PNE/PDA. (**B**) Substrate after PNE/PDA removal by NaOCl treatment. (**C**) Unpatterned PNE/PDA film. (**D**) Gold growth without the seeding step. (**E**) Seeding performed without Au^{3+} precursor. (**F**) Seeding without $NaBH_4$.

In the absence of the polymeric substrate (PNE or PDA) (**Figure S12A**), gold nanoparticles were observed on the surface as a result of secondary nucleation occurring in solution, leading to nonselective deposition. A similar outcome was obtained after removal of the PNE/PDA pattern by treatment with an aqueous NaOCl solution,[1] confirming complete polymer removal and further demonstrating the essential role of the polymeric patterns in directing nanoparticle growth (**Figure S12B**). When the polymeric layer was present but no chemical patterning was applied, the

catecholamine-based films reduced Au^{3+} ions directly, yielding randomly distributed gold nanoparticles across the surface (**Figure S12C**).

Additional control experiments were performed to elucidate the role of each step in the *in situ* gold nanoparticle synthesis. Omitting the initial seeding step resulted in randomly distributed nanoparticles (**Figure S12D**), similarly to the case in which no gold precursor was introduced during the seeding stage (**Figure S12E**). These observations confirm the crucial interaction between Au^{3+} species and the PNE/PDA surface during seeding, which is required to spatially confine nucleation events. When the $NaBH_4$ reduction step was excluded (**Figure S12F**), gold nanoparticles were still observed within the patterned regions; however, their number per patch was higher than that obtained under optimized conditions (1.8 ± 0.9 instead of 1.2 ± 0.7 for a $\Lambda = 500$ and $\varnothing = 120$ nm pattern). This result highlights the importance of the $NaBH_4$-assisted seeding step in regulating the number of active nucleation sites, thereby enabling controlled and reproducible particle densities within the patterned areas.

S6. AFM images analysis on PNE patterns after the seeding step

AFM was used to verify gold seed formation on PNE/PDA patterns (**Figure S13**). Topography images reveal localized height features confined within the polymer patches, consistent with the nucleation of metallic seeds on the polymer domains (**Figure S13A**). Complementary phase-contrast images display spatial variations in the phase signal reflecting local variations in adhesion, friction, and viscoelastic response induced by the presence of gold nuclei (**Figure S13C**). Additional insights were obtained by extracting line profiles across the same regions in both the topography and phase images (**Figure S13B,D**). The phase profile exhibits a characteristic double-peak feature separated by a minimum, which spatially coincides with the maximum observed in the corresponding topographic profile. This correlation indicates that the phase response decreases at the point of maximum topographic elevation, likely due to the altered mechanical and interfacial properties arising from the metal-polymer interface. Notably, almost all patches exhibit this characteristic contrast, with only a single patch lacking seeds, corresponding to a seed formation efficiency of ~ 97% across the analyzed field.

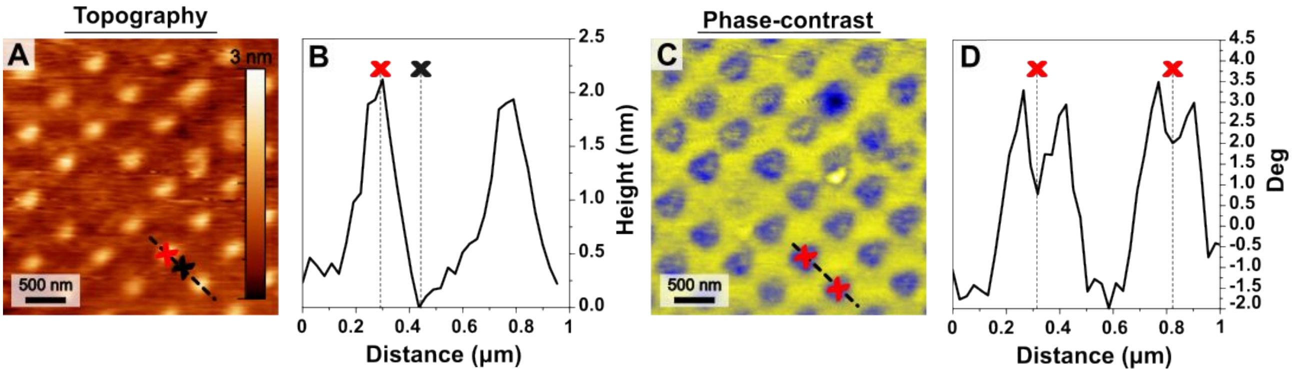

Figure S13. AFM topography images (**A**), extracted height profiles (**B**), phase-contrast images (**C**), and extracted phase profiles (**D**) performed on PNE squared patterns (Λ = 500 nm) after gold seeding step.

S7. Experimental details *in situ* growth conditions

Gold nanoparticle arrays were fabricated using a two-step seed-growth procedure optimized in this work, in which gold nucleation occurred directly on the PNE/PDA-patterned substrates, followed by a controlled growth step (**Figure S14**).

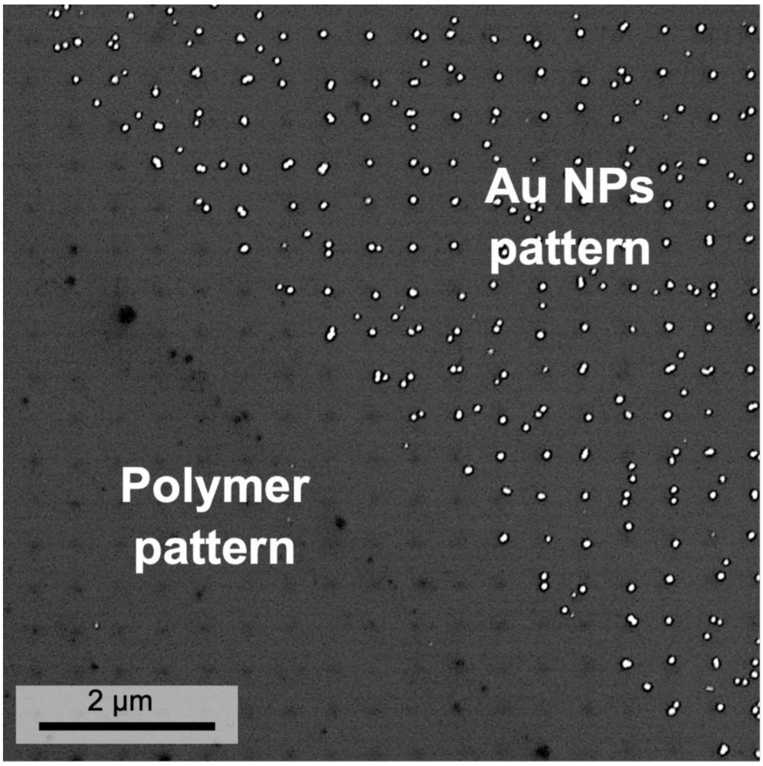

Figure S14. Representative SEM image of PNE patterns with and without *in situ* gold nanoparticle growth.

All solutions were drop-cast onto the substrates, rather than immersing the samples in order to minimize contamination from the backside of the substrates, which could otherwise induce undesired secondary nucleation. The reported solution volumes were optimized for samples with a constant surface area of 1×1 cm^2, ensuring high reproducibility and consistency across different experiments.

Seeding step

Approximately 300 µL (sufficient to fully cover the substrate surface) of an aqueous $HAuCl_4$ solution (0.01-0.5 mM) was drop-cast onto the PNE/PDA substrates and allowed to react for 5 min. The samples were then rinsed with Milli-Q water and dried under a nitrogen stream. The adsorbed Au^{3+} ions were subsequently reduced by drop-casting 300 µL of an aqueous $NaBH_4$ solution (0.6 mM), which was left to react for 10 min. After reduction, the substrates were rinsed with Milli-Q water,

kept under a static drop of water for 30 min to deactivate any residual $NaBH_4$ possibly remaining on the biopolymer-patterned surface, rinsed again with Milli-Q water, and finally dried with nitrogen.

Growth step

The growth of gold nanoparticles was performed following an established *in situ* protocol, which was optimized to limit secondary nucleation as much as possible.[2] 12 µL of a 50 mM aqueous $HAuCl_4$ solution was added to 1 mL of a 25 mM CTAB aqueous solution (acting as capping ligand and surfactant) and sonicated. Subsequently, 12 µL of a 0.1 M aqueous ascorbic acid solution was added under rapid stirring. Within approximately 5 s, the solution color faded from orange to transparent, indicating the reduction of Au^{3+} to Au^{+}. It is critical that the growth solution is vigorously stirred upon ascorbic acid addition, rapidly transferred to the substrate, and visibly clear at the moment of deposition. Immediately after the color transition, a 150 µL aliquot of the growth solution was drop-cast onto the seeded substrates and allowed to react for 1 min. The substrates were then thoroughly rinsed with Milli-Q water and dried under nitrogen. During the growth step, no further color change of the growth solution should occur. The appearance of a dark pink coloration indicates the formation of colloidal gold nanoparticles due to secondary homogeneous nucleation, which typically results from contamination of either the substrates or the growth solutions.

S8. Analysis of particle density

Here are reported the statistical analysis performed on SEM images of gold-grown patterns obtained by varying the concentration of $HAuCl_4$ solution in the seeding step.

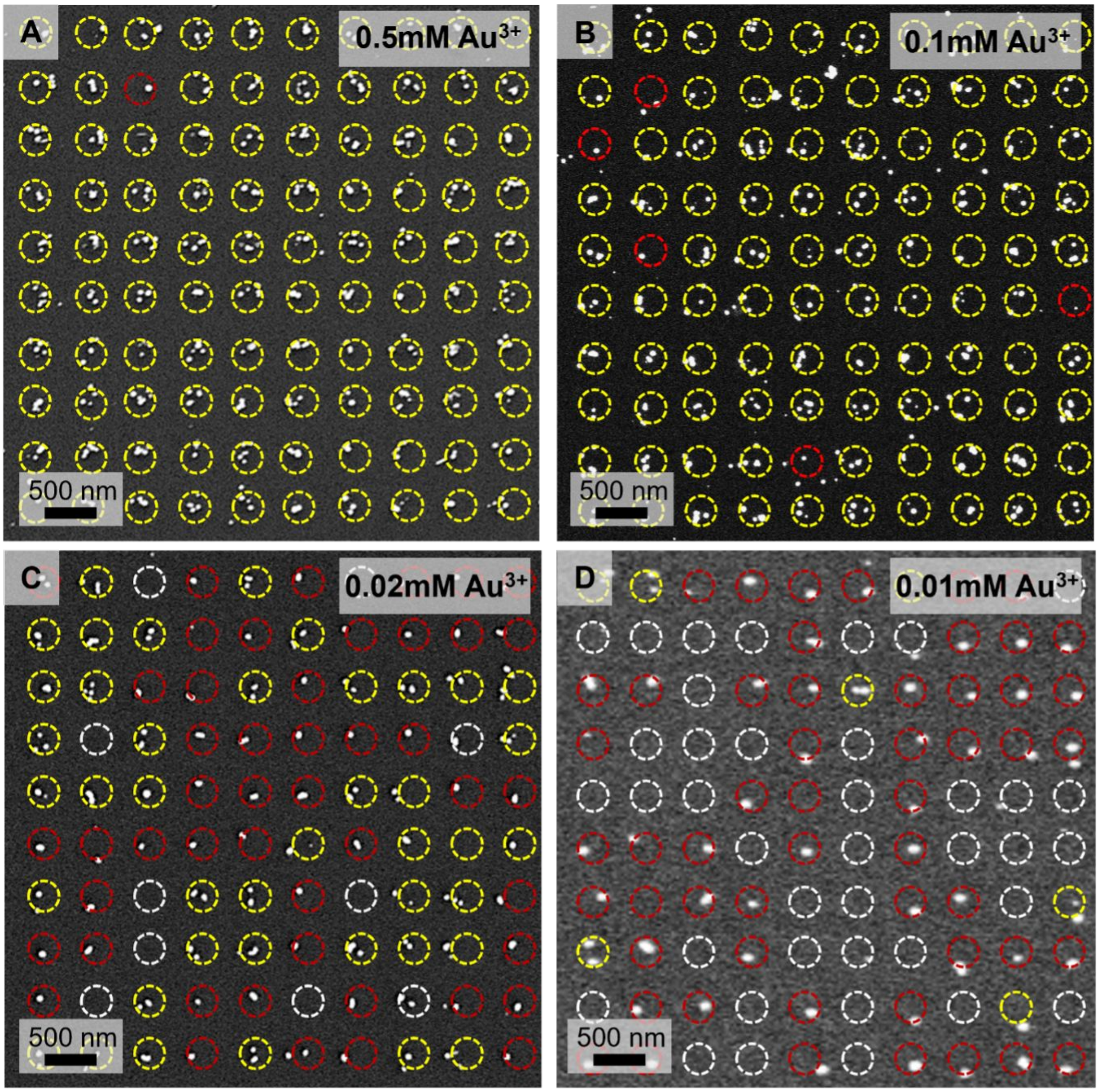

Figure S15. SEM images of gold nanoparticles grown on PNE squared patterns (Λ = 500 nm, Ø = 180 nm) obtained by varying the Au^{3+} precursor concentration during the seeding step: 0.5 mM (**A**), 0.1 mM (**B**), 0.02 mM (**C**) and 0.01 mM (**D**). Yellow circles represent regions with multiple nanoparticles; red circles represent regions with single nanoparticles and white circles represent empty regions.

For each Au^{3+} concentration condition explored, 200 polymer patterned regions were analyzed with the respective statistical analysis reported in **Table S4**.

Table S4. Statistical patterning yields for PNE squared-lattice patterns as a function of the gold precursor concentration used during the seeding step.

	**0.5 mM $HAuCl_4$**	**0.1 mM $HAuCl_4$**	**0.02 mM $HAuCl_4$**	**0.01 mM $HAuCl_4$**
% pattern yield (non-empty)	100%	100%	95%	63%
% single particle	1%	5%	47%	49%
% multiple particle	99%	95%	48%	14%
Particle/patch	4 ± 1	4 ± 2	1.6 ± 0.8	0.8 ± 0.8

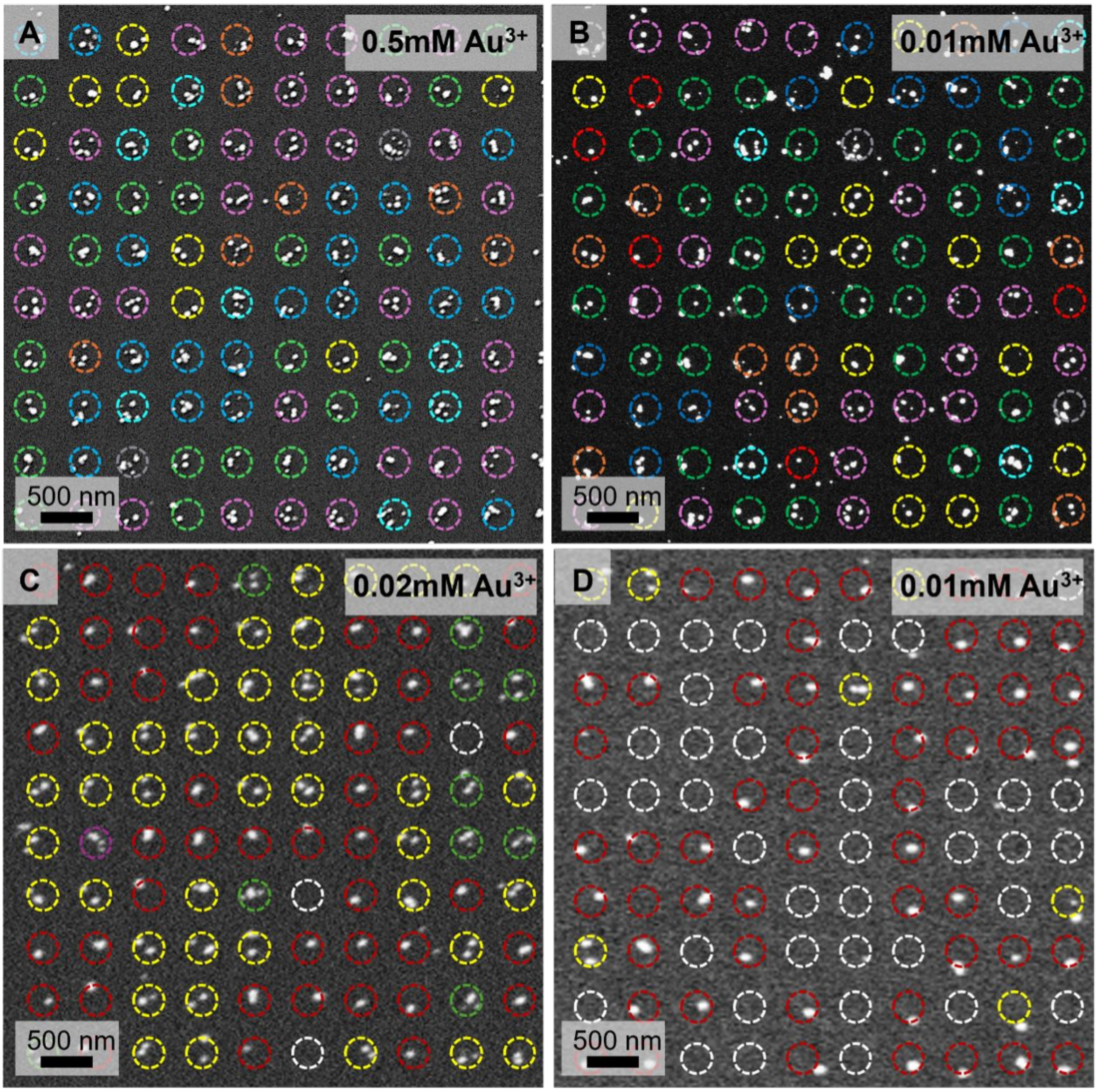

Figure S16. SEM images of gold nanoparticles grown on PNE patterns, shown in **Figure S15**, obtained by varying the Au^{3+} precursor concentration during the seeding step: 0.5 mM (**A**), 0.1 mM (**B**), 0.02 mM (**C**) and 0.01 mM (**D**). The number of particles grown in each PNE region is highlighted as follows: white circles represent empty regions, red circles represent regions with single particle, yellow circles represent regions with two particles, green circles represent regions with 3 particles, purple circles represent regions with 4 particles, blue circles represent regions with 5 particles, orange circles represent regions with 6 particles, light blue circles represent regions with 7 particles, and grey circles represent regions with 8 particles.

S9. Analysis of particle size

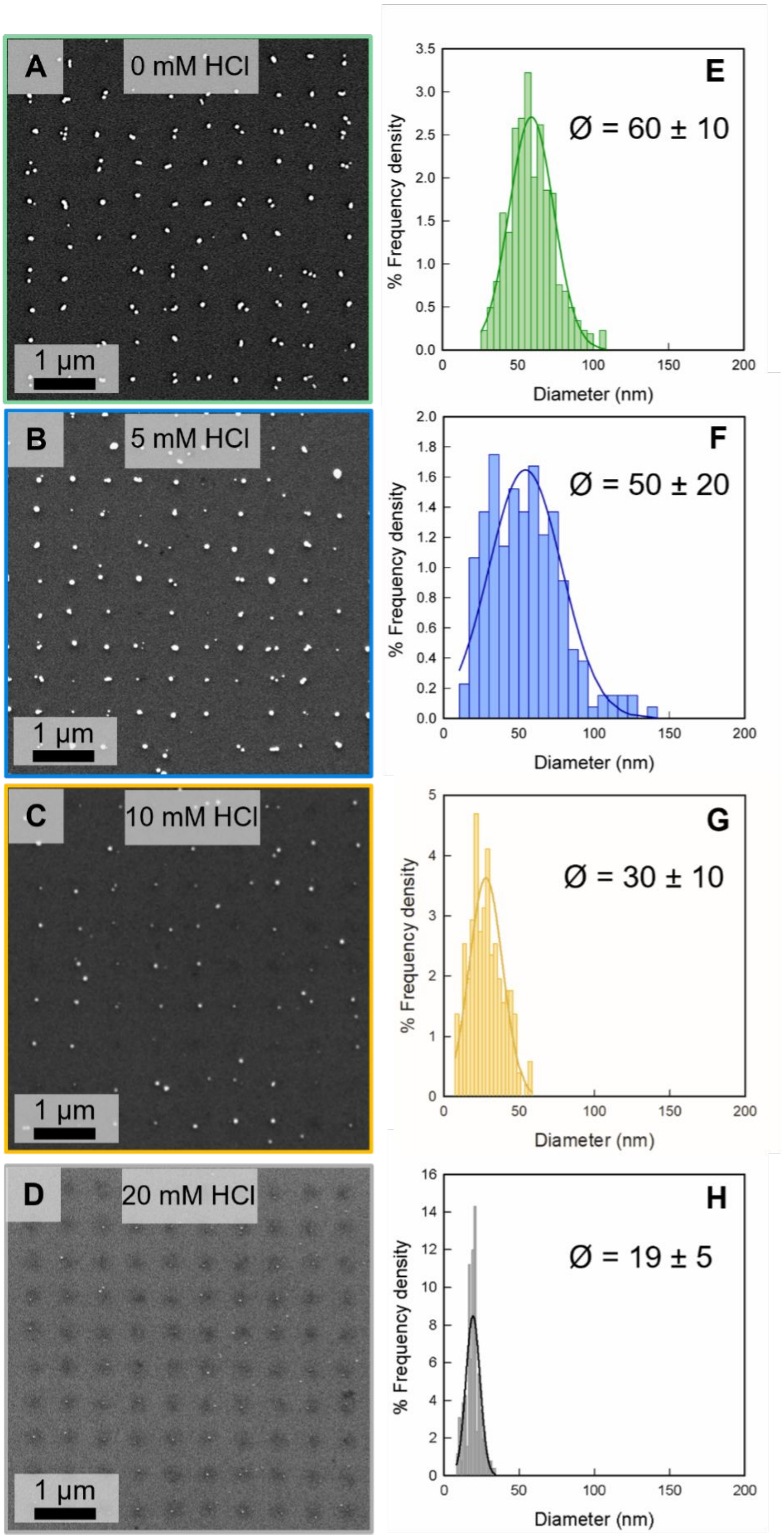

Figure S17. (**A-D**) SEM images of gold nanoparticles with different average sizes grown on square PNE patterns (Λ = 500 nm) by varying the HCl concentration in the growth solution: 0 mM (**A**), 5 mM (**B**), 10 mM (**C**), and 20 mM (**D**). (**E-H**) Size distribution obtained for each grown condition by analyzing 200 PNE patches.

S10. SEM images PDA *in situ* growth

The *in situ* protocol described in detail in the manuscript was successfully applied to produce patterned gold nanoparticles on PDA patches featuring different geometries and lattice arrangements. **Figure S18** present representative SEM micrographs of the resulting gold nanoparticles obtained on PDA-patterned substrates.

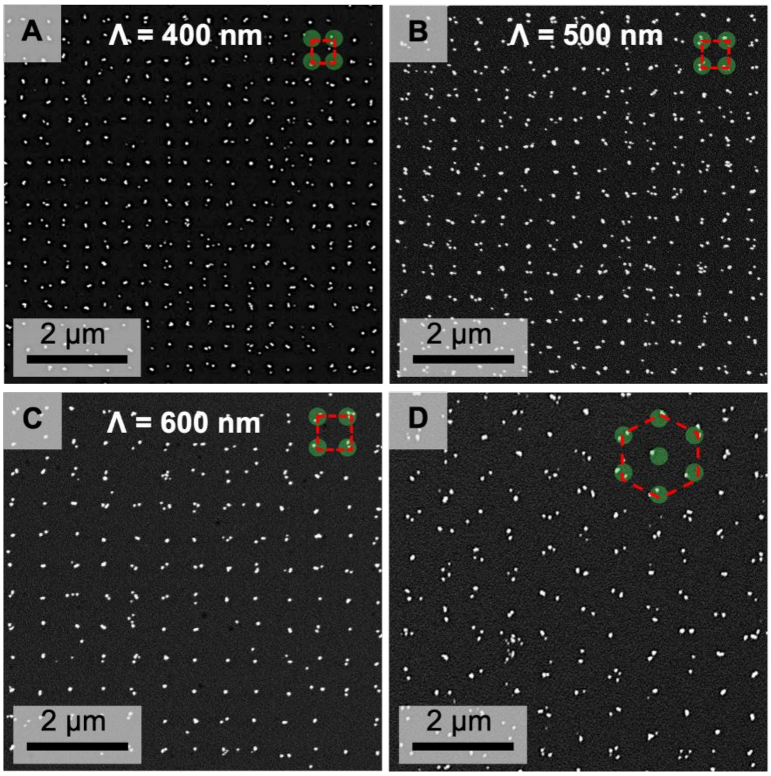

Figure S18. SEM images of patterned nanoparticle with different periodicity on PDA patterns: squared patterns of (**A**) 400 nm (Λ), (**B**) 500 nm (Λ) and (**C**) 600 nm (Λ), (**D**) hexagonal pattern.

S11. Additional SEM characterization

In order to demonstrate the successful fabrication of large-area gold nanoparticle patterns, large-scale SEM images of PDA-PNE patches featuring different geometries and lattice arrangements are reported in **Figures S19-S26**.

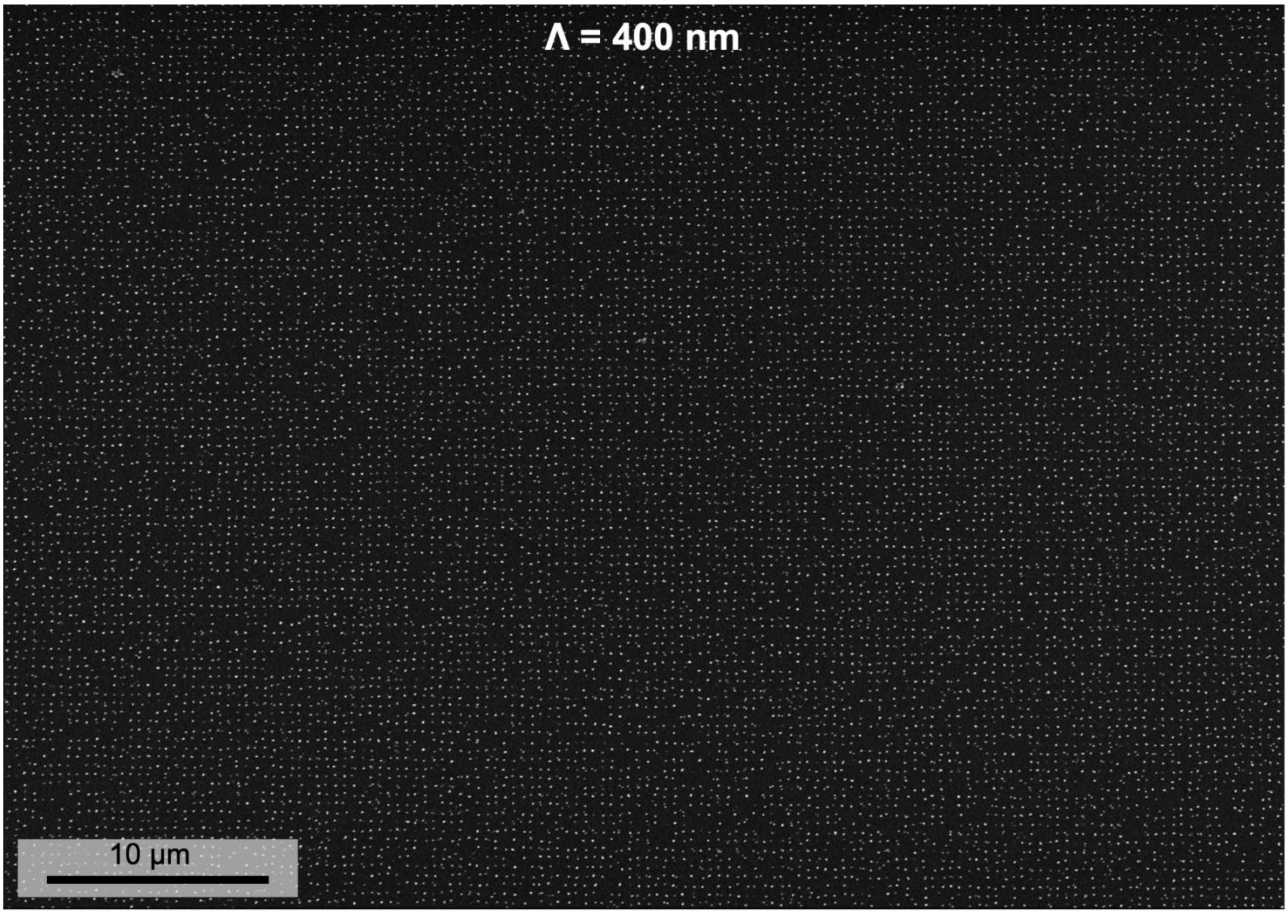

Figure S19. Large scale SEM images of patterned gold nanoparticles with squared lattice (Λ = 400 nm).

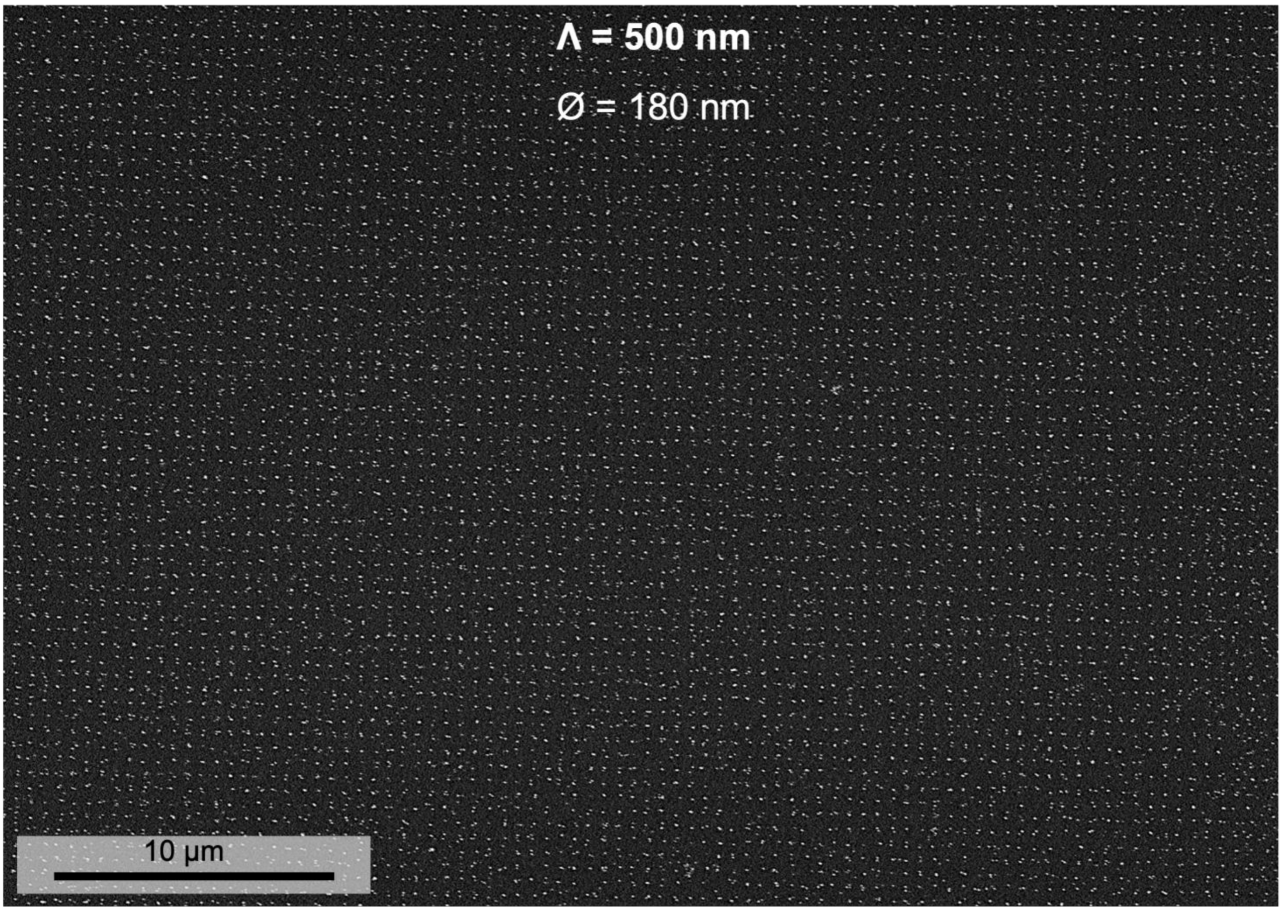

Figure S20. Large scale SEM images of patterned gold nanoparticles with squared lattice (Λ = 500 nm, Ø 180 nm).

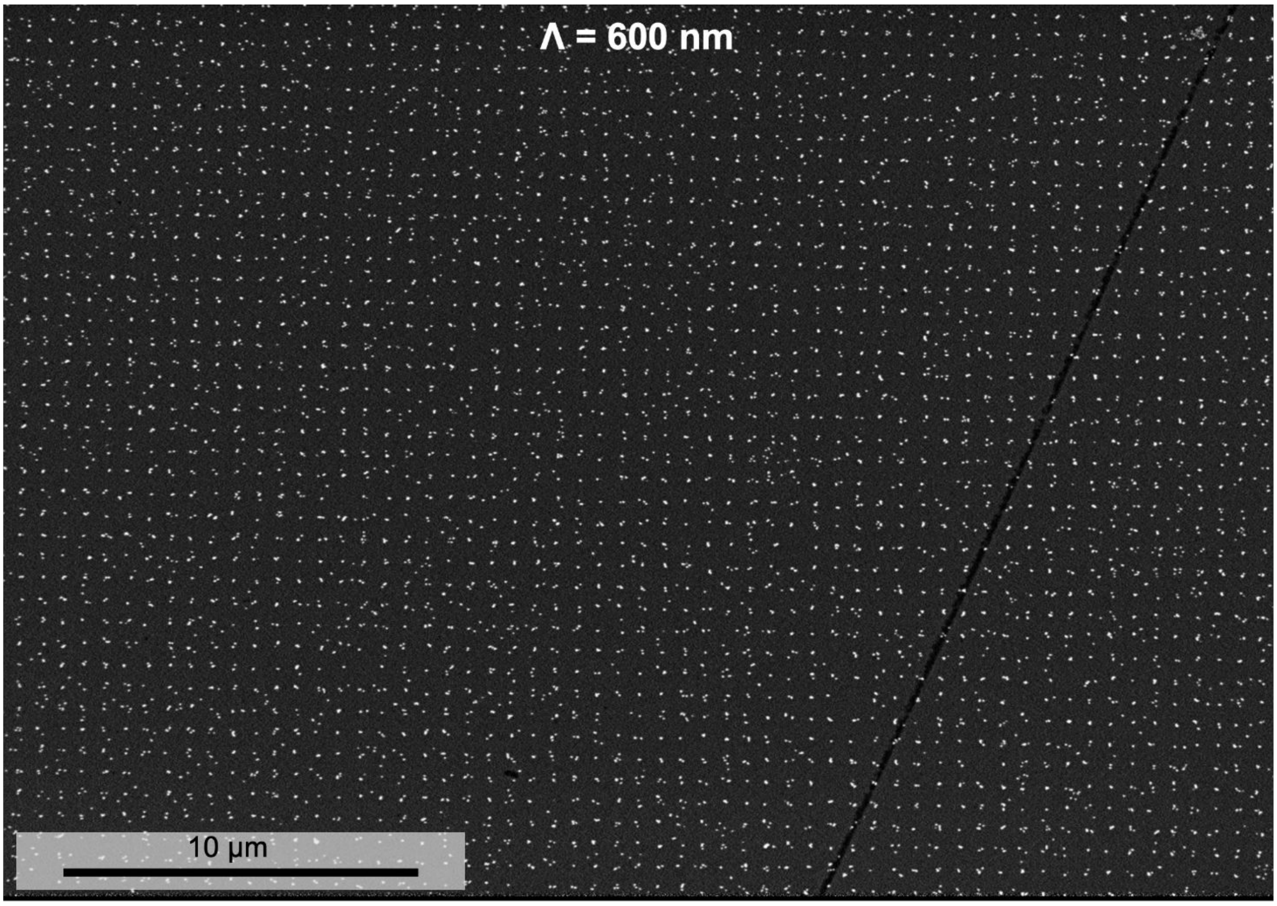

Figure S21. Large scale SEM images of patterned gold nanoparticles with squared lattice (Λ = 600 nm).

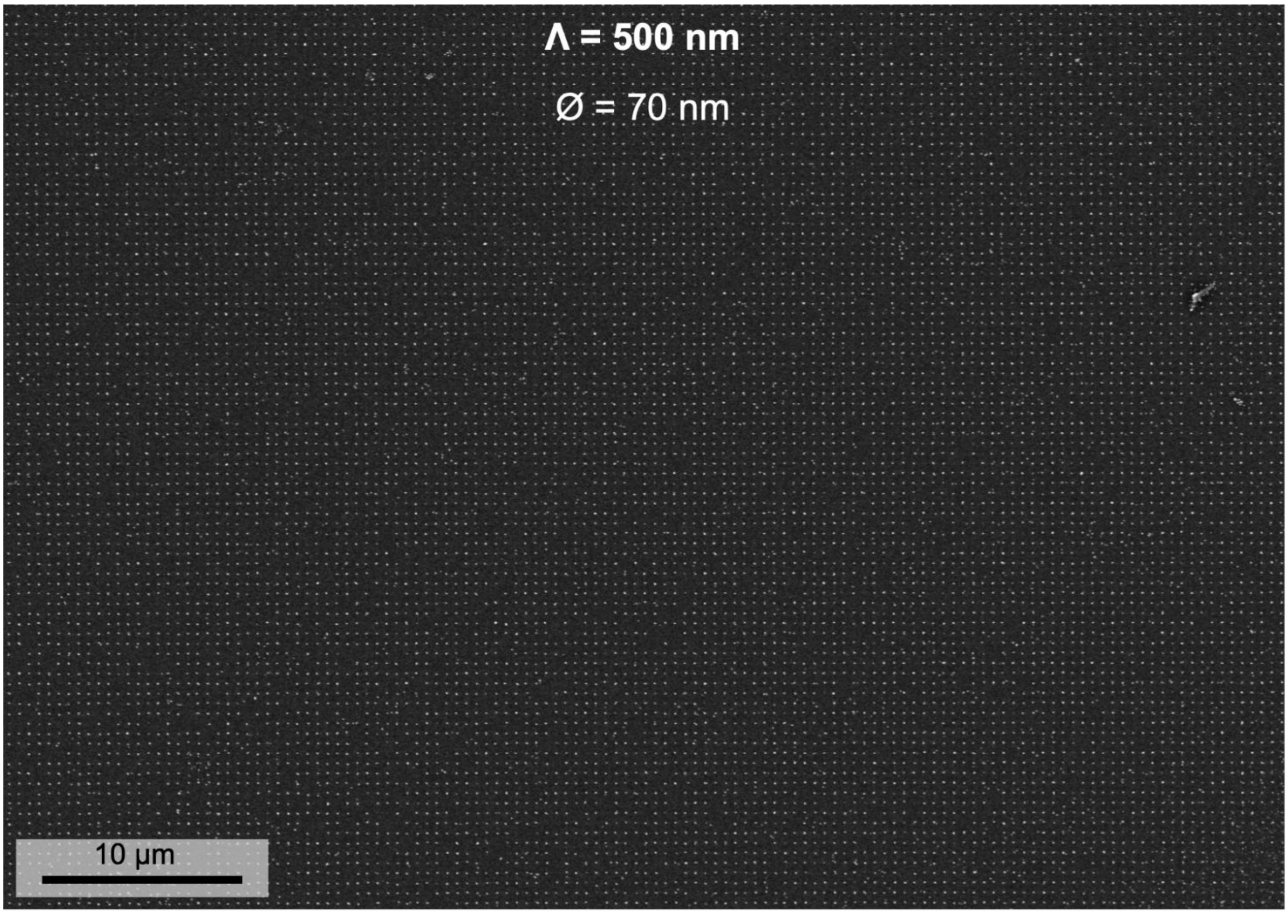

Figure S22. Large scale SEM images of patterned gold nanoparticles with squared lattice (Λ = 500 nm, Ø 70 nm).

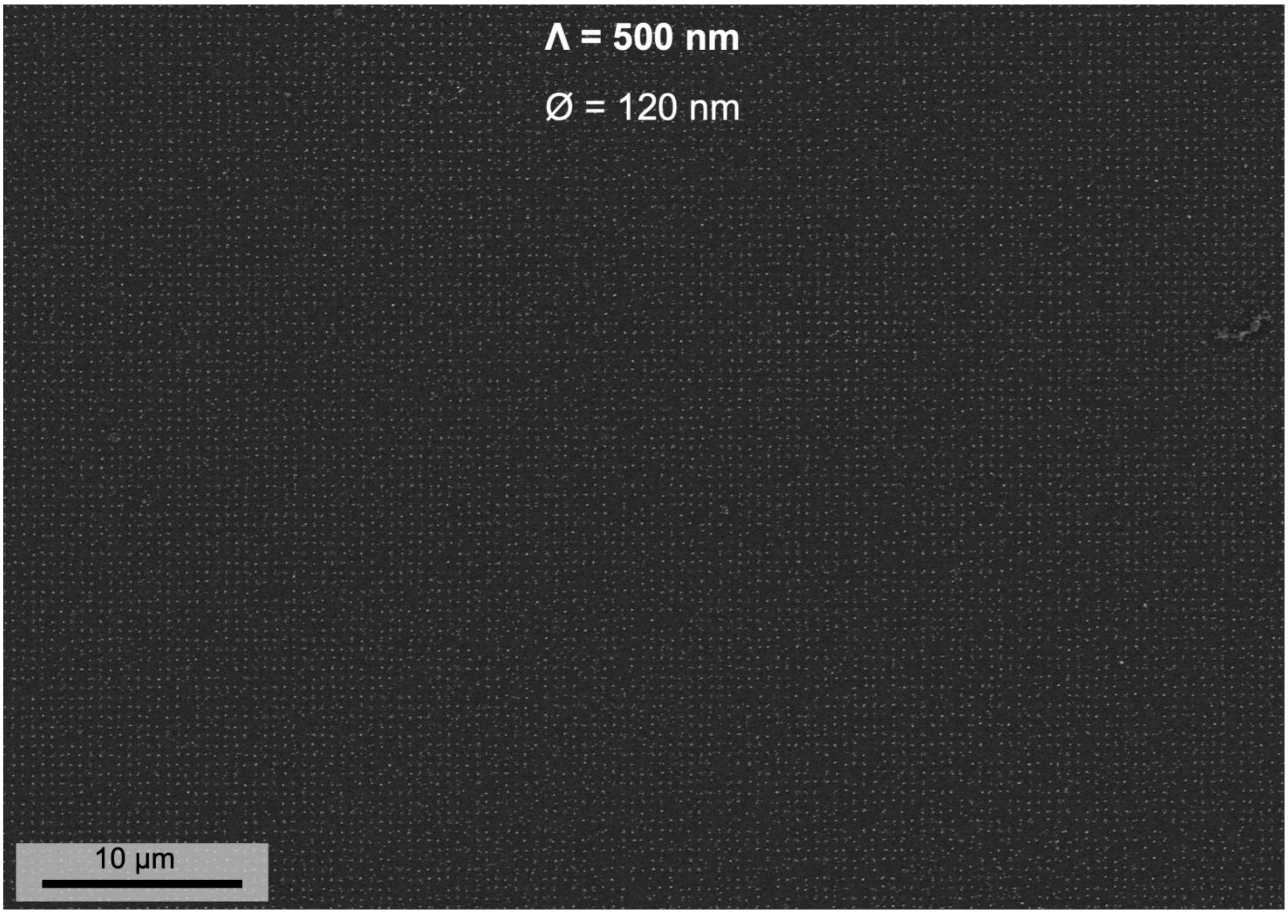

Figure S23. Large scale SEM images of patterned gold nanoparticles with squared lattice (Λ = 500 nm, Ø 120 nm).

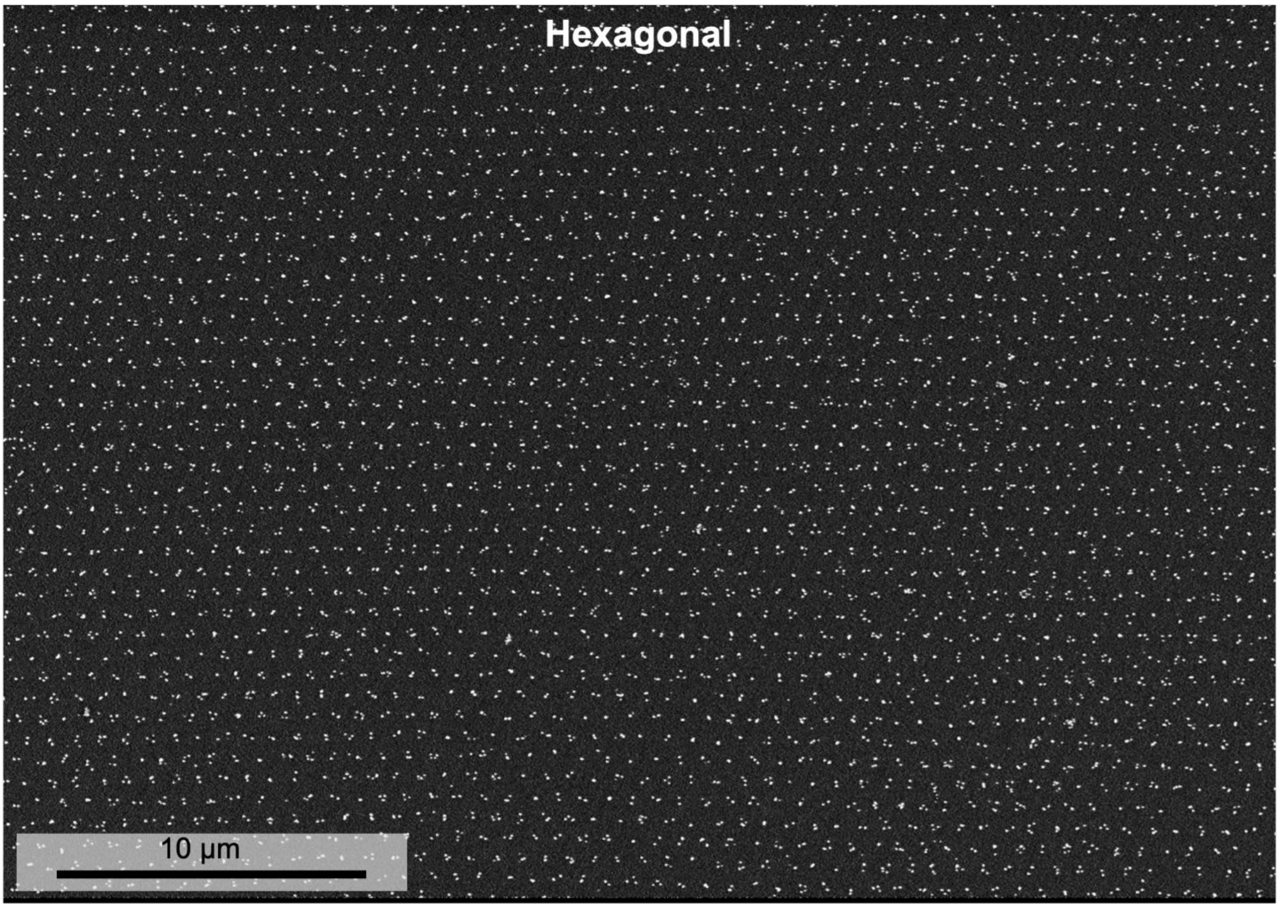

Figure S24. Large scale SEM images of patterned gold nanoparticles with hexagonal lattice.

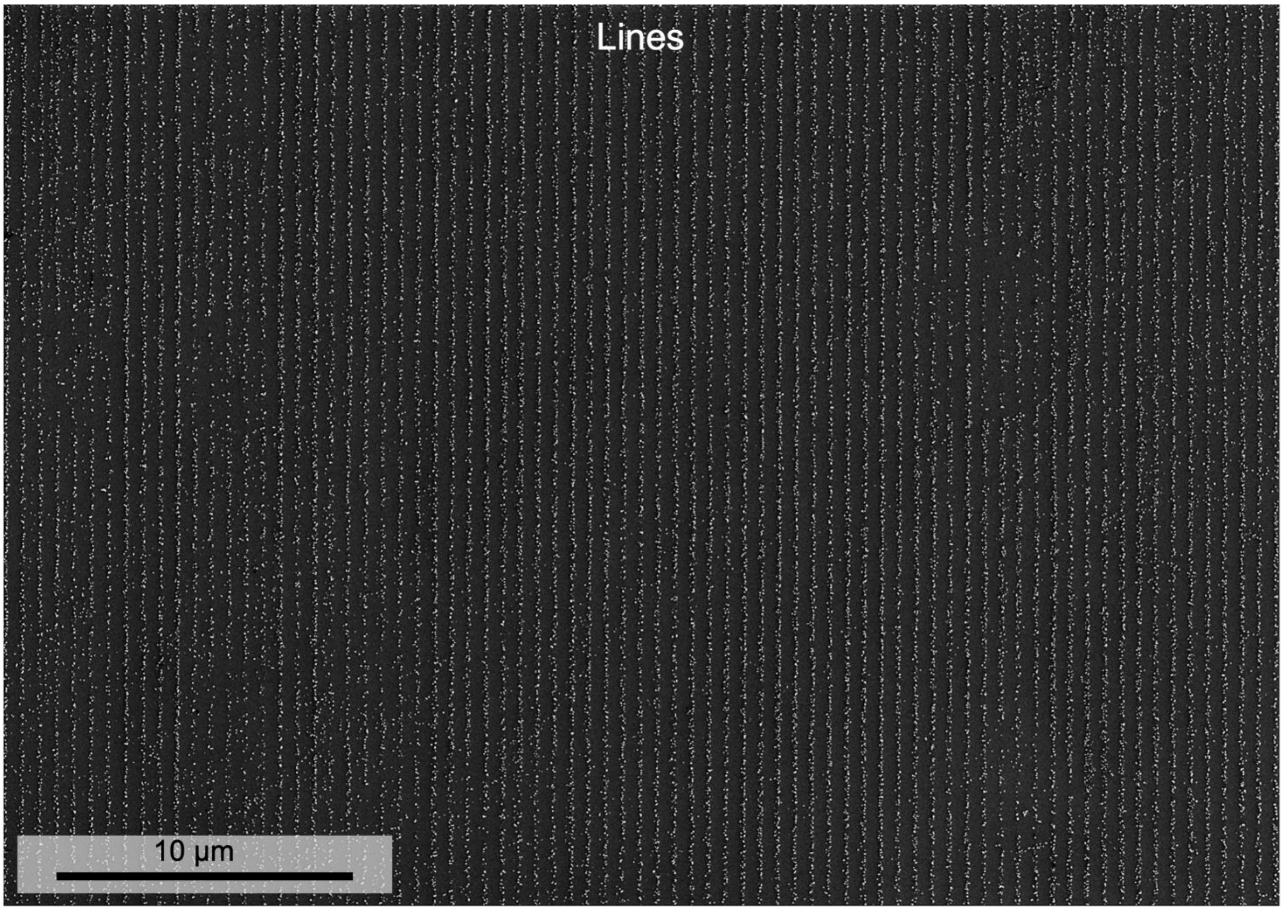

Figure S25. Large scale SEM images of patterned gold nanoparticles with line pattern.

S12. *In situ* growth on PDMS

The confined polymerization procedure and the subsequent *in situ* synthesis of gold nanoparticles were replicated on three different substrates: silicon (crystal-flat surface), glass (amorphous and non-flat surface), and PDMS (elastomeric substrate).

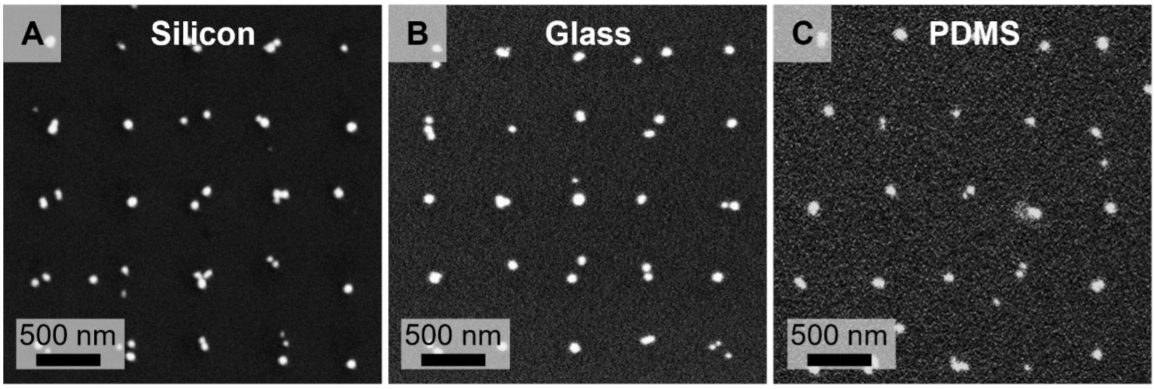

Figure S26. SEM images of patterned gold nanoparticles on different substrate materials: (**A**) silicon, (**B**) glass, and (**C**) silicon-supported flat sPDMS, with lattice parameter Λ = 600 nm.

S13. Statistical analysis of particle density by reducing polymer patches area

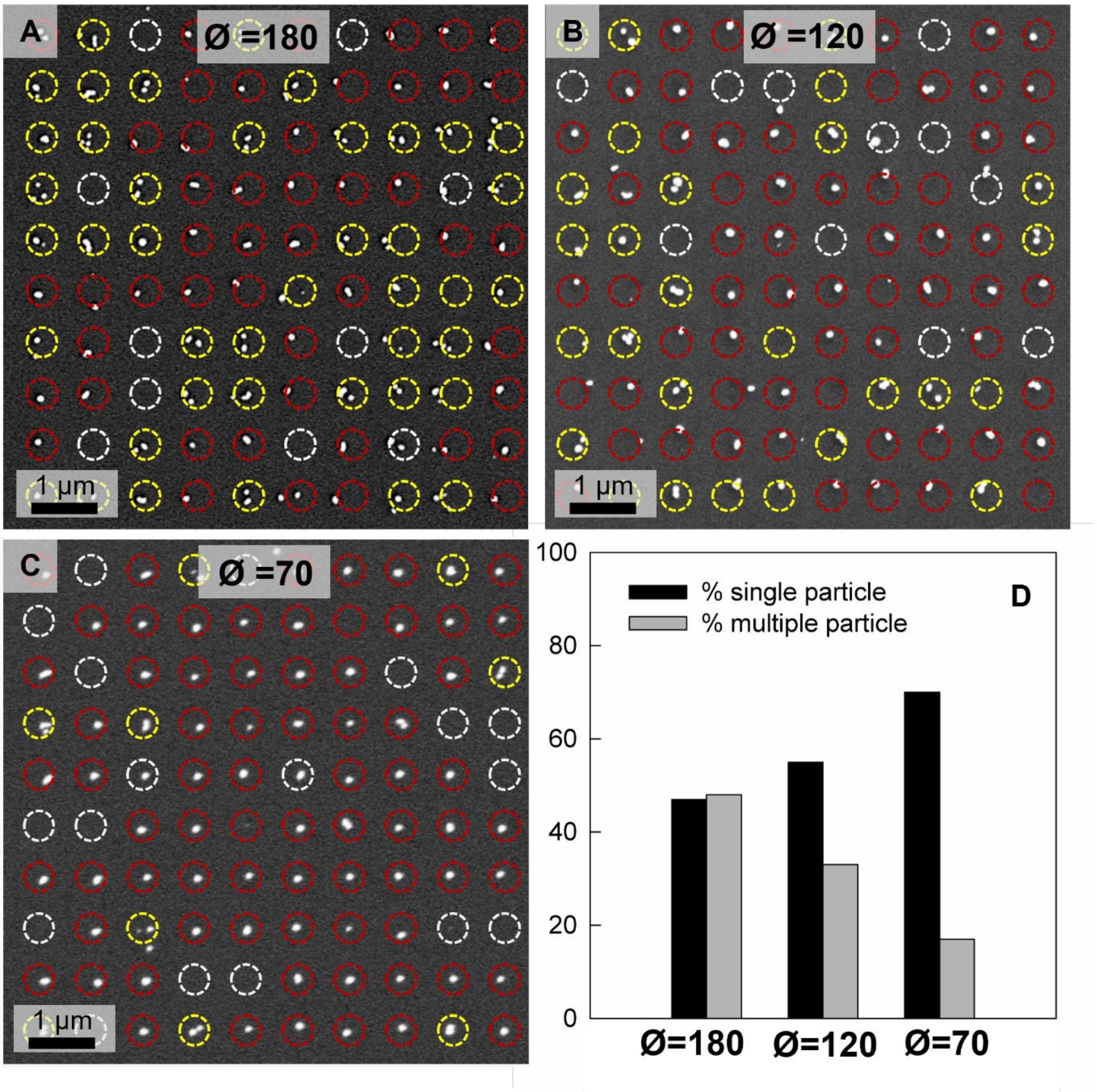

Figure S27. SEM images of gold nanoparticles grown on PNE squared patterns (Λ = 500 nm), obtained by varying the diameter of the biopolymer patches: Ø = 180 (**A**), Ø = 120 (**B**), Ø = 70 (**C**). (**D**) histogram describing the variation of the % of multiple particles grown per area (**light grey columns**) and the % of patches presenting a single nanoparticle per area (**black columns**). Yellow circles represent regions with multiple nanoparticles; red circles represent regions with single nanoparticles and white circles represent empty regions.

In **Table S5** are reported statistical data summarizing the distribution of number of particles per region as well as patterning yield obtained from the three different biopolymer feature sizes. The results were obtained by analyzing 196 polymer patches for each sample.

Table S5. Statistical patterning yields for PNE squared-lattice patterns as a function of the different feature size of polymer patches.

	**Ø 180 nm**	**Ø 120 nm**	**Ø 70 nm**
% patterned yield (non-empty)	96%	88%	87%
% particles in pattern	96%	89%	95%
% single particles	47%	55%	70%
% multiple particles	48%	33%	17%
Average n° particles/patch	1.6 ± 0.8	1.2 ± 0.7	1.0 ± 0.6

S14. Reproducibility inter-sample

To assess the reproducibility of the *in situ* growth protocol over different preparations we performed statistical analysis on three different samples of gold nanoparticles grown on PNE squared patterns (Λ = 500 nm) with biopolymer patches of 70 nm in diameter. In **Table S5** statistical data are reported summarizing the distribution of number of particles per region as well as patterning yield obtained from the three different samples. The results were obtained by analyzing 196 polymer patches for each sample.

Table S5. Statistical patterning yields for PNE squared-lattice patterns (Ø 70 nm) showing sample-to-sample reproducibility

	**Reproducibility Ø 70 nm (Average from three samples)**
% patterned yield (non-empty)	(89 ± 2) %
% particles in pattern	(96 ± 1) %
% single particles	(74 ± 8) %
% multiple particles	(16 ± 8) %
Average n° particles/patch	1.03 ± 0.06

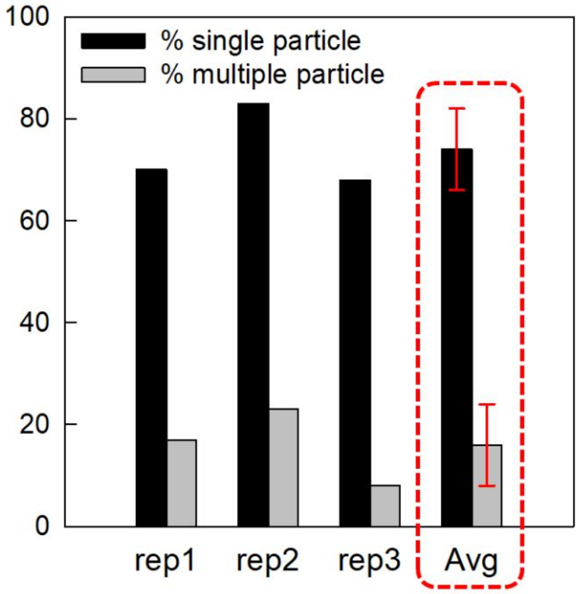

Figure S28. Histogram showing the percentage distribution of patches containing multiple nanoparticles (**light grey columns**) versus a single nanoparticle (**black columns**) for different samples with Λ = 500 nm Ø = 70 nm samples. The final two columns represent the mean values with the corresponding standard deviations.

S15. Details on optical set up and optical measurements

The experimental optical setup is shown in **Figure S29**. The hardware is controlled with a customized LabVIEW licensed program which automatizes the measurements. The plasmonic patterned area was illuminated at normal incidence using white light, produced by a Tungsten Halogen Lamp (Ocean Optics, HL-2000-HP, Florida, USA). The light was collimated on the sample and later collected using two achromatic doublet lenses (f = 50.00 mm and f = 30.00 mm). The sample holder, positioned between the two achromatic doublet lenses, was anchored on a rotational stage (Ø = 50 mm) with resonant piezoelectric motors (Thorlabs, ELL18/M) equipped with an interface board controlled by software. This allowed the automatic rotation of the illumination angle θ (from 0° to 40°) of the sample, with a resolution of 0.1°. Samples are mounted vertically and oriented along the high symmetry direction of the array on a custom-made sample holder, which enables control of the azimuthal angle φ (± 3°) alignment. The transmitted light was collected using a fiber-coupled spectrophotometer (Ocean Optics, Mayan2000) with a range of detection 380-1200 nm.

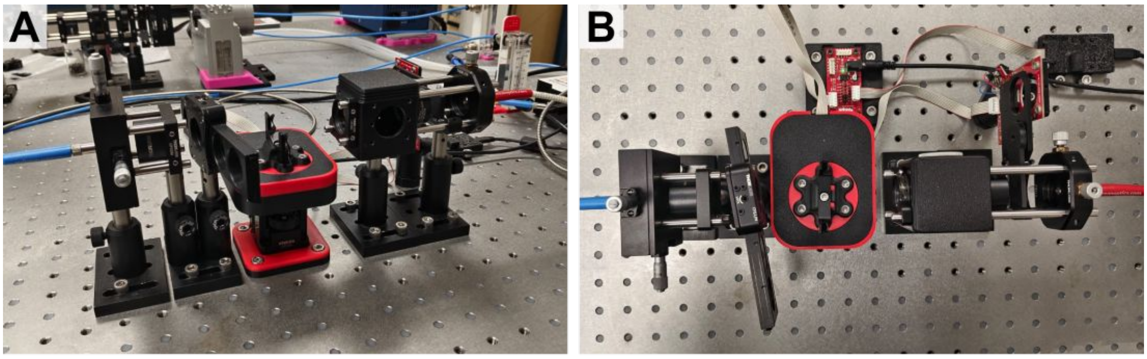

Figure S29. (**A-B**) Photo of the transmission optical setup with variable incidence angle.

In order to verify that the angular dispersion contour plots match the theoretical prediction it is necessary to expand on **Equation (1)** of the main text to take into account both illumination (*θ*) and azimuthal (*φ*) angles (see **Figure S30A**). The following equation was used to predict the diffraction lines of all the contour plots presented in the manuscript.[3,4]

$$1-\left[\left(\frac{m\lambda_{RW}}{n_{eff}\Lambda}-\frac{n_{air}}{n_{eff}}sin\theta_{inc}cos\varphi_{inc}\right)^2+\left(\frac{l\lambda_{RW}}{n_{eff}\Lambda}-\frac{n_{air}}{n_{eff}}sin\theta_{inc}cos\varphi_{inc}\right)^2\right]=0 \qquad \textbf{Equation S1}$$

where *Λ* is the lattice period, n_{eff} is the effective refractive index, *q* is the illumination angle respect to the normal direction, *φ* is the azimuthal angle, and *m* and *l* are integers indicating the diffraction orders.

The almost perfect match between experiment and theory can be verified in **Figure S30B-D**:

Figure S30. (**A**) Schematic depicting the two important angles for **Equation S1**, namely illumination (θ) and azimuthal (φ) angles. (**B-D**) Contour plots of the angular dispersion behavior between normal incidence and θ = 40º of gold plasmonic arrays grown on glass substrates with a Λ = 500 nm and a

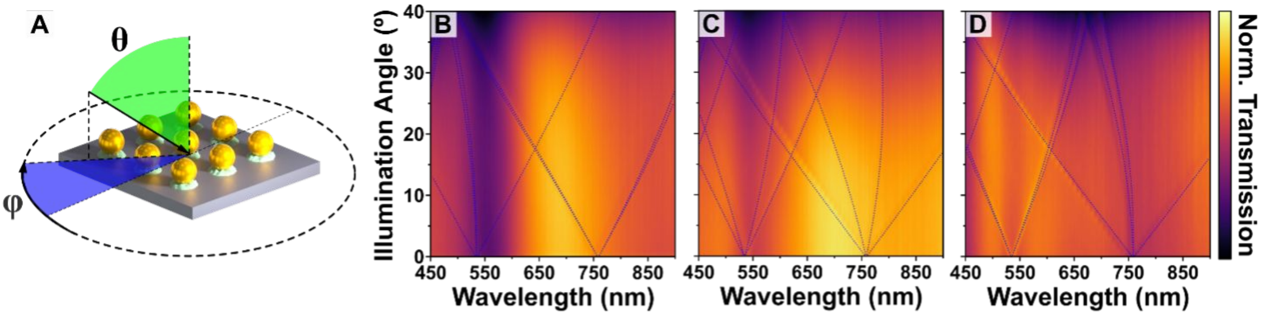

patch diameter (Ø) of 180 (**B**), 120 (**C**), and 70 nm (**D**).

Finally, 8 different normal incidence transmission spectra were collected from the same Λ = 500 and Ø = 70 nm, showing a remarkably homogenous profile (**Figure S31**).

Figure S31. Intersample variation of the transmission optical profile showing good sample homogeneity across the 1×1 cm^2 patterned area.

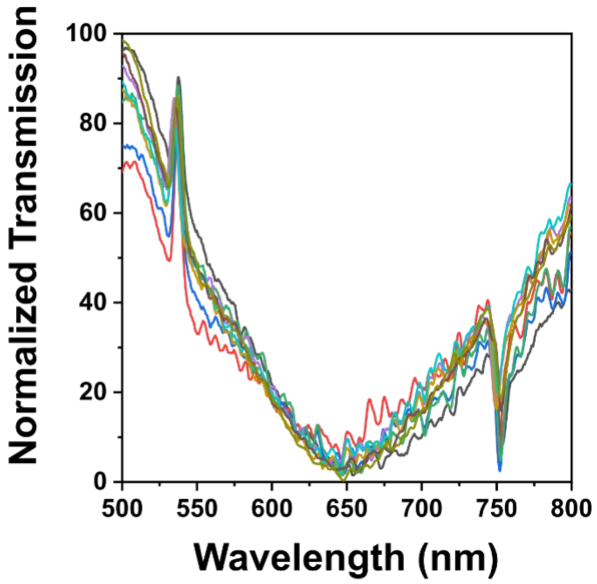

S16. Details on *Q*f calculation

Quality factor is a standard parameter used to describe the bandwidth of an optical features, which can be simply calculated as:

$$Q_{\mathrm{f}} = \frac{\lambda_r}{\Delta\lambda}$$

where λ_{r} is the resonant wavelength (center wavelength) and $\Delta\lambda$ is the bandwidth (full-width at halfmaximum, FWHM).

Before fitting the lattice resonances with a Lorentzian function, a background subtraction was performed using a customized matlab code. Background subtraction and fitting are plotted in **Figure S32** for the Λ = 500 and Ø = 70 nm sample, resulting in quality factors of 102 and 131 for the first and second diffraction order, respectively.

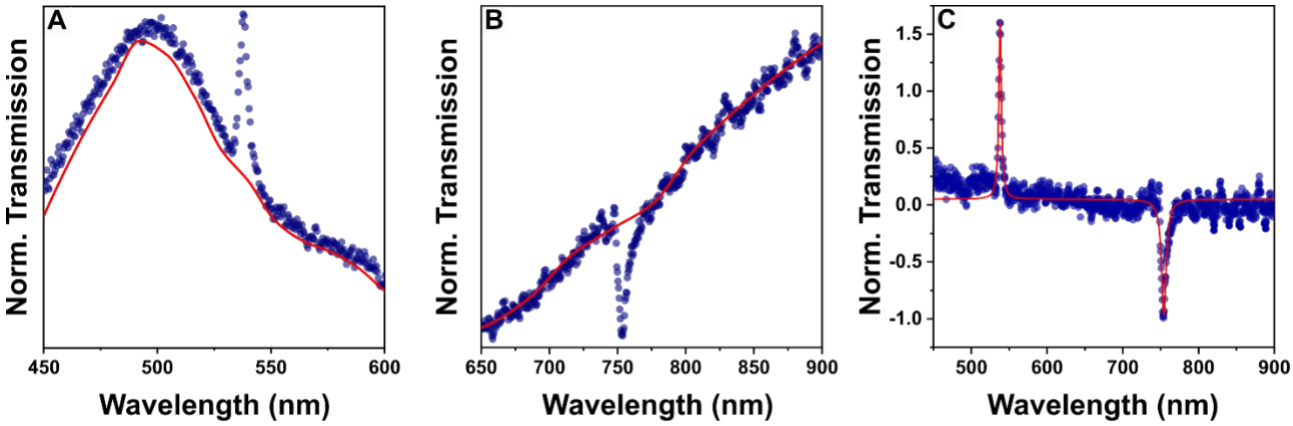

Figure S32. (**A-B**) Background subtraction on the normal incidence transmission spectrum for the Λ = 500 and Ø = 70 nm gold plasmonic array. (**C**) Fitting of the two lattice resonances with a Lorentz function.

References

1) Sestaioni, D.; Giurlani, W.; Ciacci, G.; Camagni, V.; Palladino, P.; Barucci, A.; Scarano, S. Sustainable and Effective Reconditioning of SPR Gold Chips Functionalized with Molecularly Imprinted Polynorepinephrine. *Anal. Chim. Acta* **2024**, *1321*, 343037. https://doi.org/10.1016/j.aca.2024.343037.
2) Vinnacombe-Willson, G. A.; Conti, Y.; Jonas, S. J.; Weiss, P. S.; Mihi, A.; Scarabelli, L. Surface Lattice Plasmon Resonances by Direct In Situ Substrate Growth of Gold Nanoparticles in Ordered Arrays. *Adv. Mater.* **2022**, *34* (37). https://doi.org/10.1002/adma.202205330.
3) Scarabelli, L.; Vila-Liarte, D.; Mihi, A.; Liz-Marzán, L. M. Templated Colloidal Self-Assembly for Lattice Plasmon Engineering. *Accounts Mater. Res.* **2021**, *2* (9), 816-827. https://doi.org/10.1021/accountsmr.1c00106.
4) Conti, Y.; Passarelli, N.; Mendoza-Carreño, J.; Scarabelli, L.; Mihi, A. Colloidal Silver Nanoparticle Plasmonic Arrays for Versatile Lasing Architectures via Template-Assisted Self-Assembly. *Adv. Opt. Mater.* **2023**, *11* (23). https://doi.org/10.1002/adom.202300983.
